# Bromination and conversion of tetrahydro-1H-indene to bisoxirane with a new approach: synthesis, structural characterization by spectroscopic and theoretical methods, and biological analysis supported by DFT and docking

**DOI:** 10.55730/1300-0527.3628

**Published:** 2023-10-11

**Authors:** Raşit Fikret YILMAZ, Sultan ERKAN, Salih ÖKTEN, Ahmet TUTAR, Ertan ŞAHİN

**Affiliations:** 1 Department of Fundamental Sciences, National Defence University, Turkish Naval Academy, İstanbul, Turkiye; 2 Department of Chemistry, Faculty of Science, Sivas Cumhuriyet University, Sivas, Turkiye; 3 Department of Mathematics and Science Education, Faculty of Education, Kırıkkale University, Kırıkkale, Turkiye; 4 Department of Chemistry, Faculty of Science, Sakarya University, Sakarya, Turkiye; 5 Department of Chemistry, Faculty of Science, Atatürk University, Erzurum, Turkiye

**Keywords:** Octahydroindene, dibromodiacetate, diepoxide, tetrabromo octahydroindene, density functional theory, molecular docking

## Abstract

In this study, a new method for synthesizing diepoxides is proposed. Tetrahydroindene **1** was brominated with NBS in the presence of LiClO_4_ and acetic acid, resulting in the formation of dibromodiacetate derivatives **2** and **3**. Treatment of compounds **2** and **3** with NaOH in methanol produced a mixture of diepoxides **4** and **5**. Additionally, direct bromination of tetrahydro-1H-indene yielded tetrabromo octahydroindene isomers **6** and **7**. The structures of the compounds were characterized using spectroscopic techniques such as ^1^H NMR, ^13^C NMR, APT, COSY, and XRD. The new method provides an easy and selective route to access epoxides for the synthesis of various chemicals. This study also highlights the selective formation of endo-exo and exo-exo orientations of the obtained diepoxides, distinguishing it from previous studies. The stability and properties of the stereoisomers were investigated using computational methods, revealing the most stable configurations. Reactive sites in the molecules were identified using contour diagrams and molecular electrostatic potential maps. The anticancer properties of the compounds were evaluated in silico, comparing them to 5-fluorouracil (5-FU) against several cancer cell lines. The compounds exhibited the most effective anticancer activity against MCF-7 cells, with the order of anticancer activities generally determined as **2** > **7** > **3** > **6** > **5** > **4** > 5-FU.

## 1. Introduction

Indenes are highly important in organic synthesis due to their prevalence in the structural ring motifs of bioactive pharmaceutical agents, functional materials, and natural products. They also serve as valuable ligands in olefin polymerization reactions [1].

Epoxides are versatile functional groups with high reactivity, finding extensive use in various applications. They serve as valuable precursors for the synthesis of biologically active compounds, especially antibiotics and tumor inhibitors, owing to their relative stability. The catalytic epoxidation of indene has garnered significant interest as it offers convenient routes to important bioactive chemicals [2]. Epoxides are crucial structural moieties in numerous natural products like triptolide, epothilones, and cryptophycin, contributing to their biological activities. Furthermore, epoxides play a vital role as intermediates in the biosynthesis of various natural products, including brevetoxin B, monensin, and glabrescol [3]. Commercially, epoxides serve as building blocks for epoxy resins, paints, surfactants, perfume materials, plasticizers, and sweeteners [4,5]. Consequently, researchers have focused on developing efficient synthetic methods for epoxidation [3].

Epoxidation is an indispensable reaction type for the chemical industry as it enables the conversion of olefins into oxygenated molecules [6]. The unique polarity and strain of the three-membered epoxide rings allow them to participate in various reactions with a wide range of reagents, including electrophiles, nucleophiles, acids, bases, and radicals [7].

Numerous methods have been developed for the preparation of epoxides. Typically, epoxides are formed by reacting an olefin with an oxidizing agent in the presence of a catalyst [8]. The imidazole system has demonstrated high catalytic activity and selectivity in the epoxidation of cycloaliphatic alkenes using 30% aqueous H_2_O_2_ as the sole oxidant [9]. Other methods for epoxidation reactions involve the use of peracids such as peracetic acid or monoperoxyphthalic acid [10,11].

In some cases, stereoselective epoxide derivatives can be obtained through base-induced dehydrobromination of naphthalene and anthracene in the presence of silver ions [12–14]. Although bisbromoacetate was used as a starting compound for syn- and anti-diepoxides, the efficiency was relatively low (5%–10%) [15].

Racemic mixtures of epoxides hold biological significance due to their kinetic solubility and various chemical and biocatalytic properties [16,17]. They readily react with nucleophilic groups such as epoxides, halides, carbon, nitrogen, oxygen, or sulfur. Epoxides have been found in the structures of certain carcinogens, but they are also present in therapeutically interesting molecules [18–20]. Additionally, epoxides serve as valuable building blocks in the synthesis of numerous drugs, particularly as alkylating and cross-linking agents. Many anticancer substances incorporate epoxide functionalities [21]. Consequently, their potential significance in biological processes is high. Studies in the literature indicate that epoxy compounds can inhibit damaged DNA and exhibit toxic effects on cancer cells [21]. As a result, the undeniable biological importance of epoxides positions them as a leading compound class in drug design.

Lithium perchlorate (LiClO_4_) has garnered significant attention as a catalyst due to its notable rate acceleration and enhanced selectivity [22]. The Lewis acidic nature of the lithium ion contributes to improved reactivity and endo-exo selectivity in bicyclic systems [23], particularly at room temperature in reaction media [24–26].

In this study, we present a new synthesis pathway for the production of diepoxy indene derivatives and tetrabromides 6 and 7 under mild reaction conditions. Novel dibromodiacetates 2 and 3 were transformed into diepoxides 4 and 5 through their interaction with organic solvents and bases. Detailed discussions on the reaction mechanisms of all products were provided and the compounds were characterized using spectral data and chemical transformations. Additionally, we employed computational chemistry methods to investigate all experimentally synthesized compounds (**2**–**7**) at a molecular level due to the fact that compounds 2–7 are natural products and can be used in many different fields, such as for pharmaceuticals or organic light-emitting diodes. Calculations were performed using conceptual density functional theory at the B3LYP/6-31G (d) level, leading to the determination of optimized structures for the relevant compounds. Stability analyses were conducted for stereoisomeric molecules 2, 3, 4, 5, 6, and 7. A comparison between the experimental spectroscopic data and the spectroscopic results obtained from the calculations was performed. Furthermore, we aimed to explore the molecular electrostatic potential (MEP) maps, contour diagrams, and anticancer properties of compounds **2**–**7** in comparison to 5-fluorouracil (5-FU). We examined how the anticancer effects varied with changes in stereoisomers and structural properties.

## 2. Experimental

### 2.1. Materials

All chemicals and solvents utilized in the synthesis process were obtained from Merck Chemical Company (Darmstadt, Germany). Thin-layer chromatography was conducted on Merck silica F254 plates with a thickness of 0.255 mm and the spots were visualized using UV light at a wavelength of 254 nm. Column chromatography was performed using Merck 60 silica gel with a mesh size of 70–230. The melting points were determined using an MPM-H1 capillary melting point apparatus (Flinn Scientific, Batavia, Illinois, USA). Solvents were concentrated under reduced pressure. FT-IR spectra were recorded using a Shimadzu Prestige-21 (200 VCE) FT/IR instrument (Shimadzu Corporation, Kyoto, Japan). NMR spectra were obtained using an Infinity Plus spectrometer (Varian, Santa Clara, CA, USA) operating at 300 MHz for ^1^H NMR and 75 MHz for ^13^C NMR. Elemental analysis was conducted using a Vario MICRO Cube instrument (Elementar, Langenselbold, Germany).

### 2.2. Synthesis of dibromodiacetate 2 and 3

A mixture of acetic acid (CH_3_COOH, 15 mL), 3a,4,7,7a-tetrahydro-1*H*-indene (THI, **1**) (0.84 g, 7 mmol), NBS (3.57 g, 20 mmol), and LiClO_4_ (0.21 g, 2 mmol) was added to a two-necked reaction flask (50 mL) covered with aluminum foil and stirred at room temperature for 48 h. The reaction mixture was then transferred to a flask and a slow and careful addition of saturated NaHCO_3_ solution (100 mL) was performed. The resulting mixture was extracted with diethyl ether (3 × 35 mL). The organic phase was dried over Na_2_SO_4_ and the solvent was evaporated under reduced pressure. A brown viscous mixture (2.23 g) was obtained, which showed two products according to ^1^H NMR analysis. The crude product was purified by column chromatography (hexane/EtOAc, 10/1). The first product (**2**) was obtained from the fractions between 250 and 500 mL. The product of dibromodiacetate **3** was obtained from fractions between 600 and 900 mL. The identification of this product was confirmed by ^1^H NMR. The white, dense dibromodiacetate **3** product started to crystallize within a few hours at room temperature. A mixture of hexane and ether was then added to the dibromodiacetate **3** product for further crystallization. The mixture was kept at room temperature overnight and subsequently placed in the refrigerator. After one night, clear white needle-shaped crystals had formed.

***Rel*-(1R,2R,5R,6R)-1,6-dibromoctahydro-1*H*-indene-2,5-diyl diacetate (2):** Oily yellow liquid; 1.08 g, isolated yield 39%; ^1^H NMR (CDCl_3_, 300 MHz, ppm) δ_H_ 5.15–5.09 (m, 1H), 4.88–4.82 (m, 1H), 4.13–4.04 (m, 2H), 2.51–2.32 (m, 2H), 2.23–2.15 (m, 1H), 2.14–2.00 (m, 3H), 1.96 (s, 3H), 1.94 (s, 3H), 1.72–1.62 (m, 1H), 1.38–1.29 (m, 1H); ^13^C NMR (CDCl_3_, 75 MHz, ppm) δ_C_ 170.0 (C), 169.8 (C), 82.0 (CH), 72. 8 (CH), 56.4 (CH), 48.6 (CH), 45.3 (CH), 35.2 (CH_2_), 33.9 (CH_2_), 33.7 (CH), 27.2 (CH_2_), 21.3 (CH_3_), 21.2 (CH_3_); FT IR (cm^−1^) *v* 2941, 1732, 1435, 1365, 1228, 1209, 1043, 1022, 906, 727, 648; Anal. calcd for C_13_H_18_Br_2_O_4_ (395.96): C, 39.22; H, 4.56; Found: C, 39.28; H, 4.49.

***Rel*-(1R,2R,5S,6S)-1,6-dibromoctahydro-1*H*-indene-2,5-diyl diacetate (3):** Colorless needle crystals; mp: 85–86 °C; 1.03 g, isolated yield 37%; ^1^H NMR (CDCl_3_, 300 MHz, ppm) δ_H_ 5.29–5.23 (m, 1H), 4.90–4.82 (m, 1H), 4.13–3.99 (m, 2H), 2.67–2.60 (m, 1H), 2.53–2.44 (m, 1H), 2.33–2.20 (m, 2H), 2.19–2.12 (m, 2H), 2.09 (s, 3H), 2.08 (s, 3H), 1.74–1.35 (m, 2H); ^13^C NMR (CDCl_3_, 75 MHz, ppm) δ_C_ 170.3 (C), 170.2 (C), 82.2 (CH), 75.2 (CH), 53.1 (CH), 48.3 (CH), 47.6 (CH), 37.1 (CH_2_), 35.2 (CH), 34.8 (CH_2_), 33.7 (CH_2_), 21.3 (CH_3_), 21.2 (CH_3_); FT IR (cm^−1^) *v* 2983, 2935, 2868, 1737, 1448, 1427, 1377, 1361, 1303, 1273, 1228, 1192, 1155, 1130, 1114, 1043, 1024, 975, 954, 927, 896, 858, 827, 815, 781, 734, 709, 640; Anal. calcd for C_13_H_18_Br_2_O_4_ (395.96): C, 39.22; H, 4.56; Found: C, 39.26; H, 4.60.

### 2.3. Epoxidation reactions

#### 2.3.1. Epoxidation of dibromoacetate mixture

The mixture of dibromodiacetate (**2** and **3**) (0.6 g) was dissolved in 25 mL of methyl alcohol (CH_3_OH) in a two-necked reaction flask. NaOH (1.5 g) was added and the reaction mixture was stirred at room temperature for 48 h. The resulting mixture was transferred to a flask and pure water (25 mL) was added. The mixture was extracted with dichloromethane (CH_2_Cl_2_) (3 × 35 mL). The organic phase was dried over Na_2_SO_4_, and the solvent was evaporated under reduced pressure. ^1^H NMR analysis showed a mixture of products. The crude product was purified by column chromatography. The mixture of products was applied to a short silica gel chromatography column eluting with hexane. The first eluent (70–150 mL) yielded diepoxide 4 (98 mg, isolated yield 43%) in hexane. Diepoxide 5 was obtained in the second eluent between 180 and 220 mL (92 mg, isolated yield 40%).

Since the dibromodiacetate mixture (2 and 3) was isolated separately, the diepoxide reaction was performed for each isomer. As a result, diepoxide 4 was obtained from the reaction of dibromodiacetate 2 and diepoxide 5 from the reaction of dibromodiacetate 3.

#### 2.3.2. Synthesis of diepoxide 4

Dibromodiacetate 2 (0.50 g, 1.16 mmol, 1 eq.) was dissolved in CH_3_OH (15 mL) in a two-necked reaction flask. NaOH (0.13 g, 3.14 mmol, 2.5 eq.) was added to the reaction mixture and stirred at room temperature for 44 h. The reaction mixture was then taken up in a flask and pure water (25 mL) was added. The mixture was extracted with dichloromethane (CH_2_Cl_2_) (3 × 35 mL). The organic phase was dried over Na_2_SO_4_ and the solvent was removed under reduced vacuum. The crude product was purified with a short silica gel column, yielding diepoxide 4 (0.12 g, 64% yield).

***Rel*-(1aR,2aR,3aS,5aS)-octahydro-1a*H*-indeno[1,2-b:5,6-b’]bis(oxirene)/(3,4,7,8-diepoxybicyclo[4.3.0]nonane) (4):** Brown oily liquid; 90 mg, isolated yield 64%; ^1^H NMR (CDCl_3_, 300 MHz, ppm) δ_H_ 3.44–3.42 (m, 1H), 3.29–3.27 (m, 1H), 3.11–3.07 (m, 1H), 3.03–2.98 (m, 1H), 2.28–2.15 (m, 2H), 2.03–1.89 (m, 3H), 1.68–1.55 (m, 2H), 1.27–1.15 (m, 1H); ^13^C NMR (CDCl_3_, 75 MHz, ppm) δ_C_ 62.2 (CH), 59.2 (CH), 51.4 (CH), 51.2 (CH), 36.6 (CH), 33.8 (CH_2_), 32.1 (CH_2_), 31.6 (CH), 24.9 (CH_2_); FT IR (cm^−1^) *v* 3007, 2926, 2862, 1456, 1419, 1398, 1340, 1327, 1288, 1226, 1165, 1116, 1080, 1041, 1026, 987, 906, 819, 796, 761, 655; Anal. calcd for C_9_H_12_O_2_ (152.02): C, 71.03; H, 7.95; Found: C, 71.18; H, 8.01.

#### 2.3.3. Synthesis of diepoxide 5

Dibromodiacetate **3** (0.50 g, 1.16 mmol, 1 eq.) was dissolved in CH_3_OH (15 mL) in a two-necked reaction flask. NaOH (0.13 g, 3.14 mmol, 2.5 eq.) was added to the reaction mixture and stirred at room temperature for 44 h. The reaction mixture was then taken up in a flask and pure water (25 mL) was added. The mixture was extracted with dichloromethane (CH_2_Cl_2_) (3 × 35 mL). The organic phase was dried over Na_2_SO_4_ and the solvent was evaporated under reduced pressure. The crude product was purified by column chromatography in hexane, yielding diepoxide **5** (0.11 g, 56% yield).

***Rel*-(1aR,2aS,3aS,5aS)-octahydro-1a*H*-indeno[1,2-b:5,6-b’]bis(oxirene)/(3,4,7,8-diepoxybicyclo[4.3.0]nonane) (5):** Brown oily liquid; 50 mg, isolated yield 56%; ^1^H NMR (CDCl_3_, 300 MHz, ppm) δ_H_ 3.81–3.77 (m, 1H), 3.68–3.66 (d, 1H), 3.46–3.36 (d, 1H), 3.26–3.10 (d, 1H), 2.21–2.14 (m, 1H), 2.10–2.03 (m, 1H), 1.95–1.85 (m, 2H), 1.79–1.69 (m, 2H), 1.55–1.44 (m, 2H); ^13^C NMR (CDCl_3_, 75 MHz, ppm) δ_C_ 66.2 (CH), 62.7 (CH), 60.8 (CH), 56.7 (CH), 36.4 (CH), 34.1 (CH_2_), 30.8 (CH_2_), 26.4 (CH), 26.3 (CH_2_); FT IR (cm^−1^) *v* 3022, 2927, 1739, 1647, 1558, 1541, 1463, 1436, 1363, 1340, 1296, 1228, 1099, 1024, 977, 893, 846, 748, 653; Anal. calcd for C_9_H_12_O_2_ (152.02): C, 71.03; H, 7.95; Found: C, 71.12; H, 7.99.

### 2.4. Synthesis of tetrabromo octahydroindenes (6 and 7)

To a solution of 3a,4,7,7a-tetrahydro-1H-indene (THI, 1, 1.2 g, 10 mmol) in CH_2_Cl_2_ (15 mL), a solution of bromine (3.2 g, 20 mmol, 2 eq.) in CH_2_Cl_2_ (5 mL) was added slowly over a period of 10 min in the dark at room temperature. After completion of the reaction (when the bromine was completely consumed, 10 min), the solvent of the resulting mixture was removed under reduced pressure. ^1^H NMR analysis displayed two products. The crude product was purified by column chromatography (hexane/EtOAc, 10/1). The first product (**6**) was obtained in the fractions collected between 120 and 450 mL. The second product, tetrabromide **7**, was obtained in the fractions collected between 500 and 700 mL. The white and dense tetrabromide **6** product began to crystallize within a few hours at room temperature. After one night, clear white needle-shaped crystals had formed. The solvent of tetrabromide **7** was removed under reduced pressure, resulting in the formation of a colorless oil product.

***Rel*-(1R,2R,5S,6S)-1,2,5,6-tetrabromooctahydro-1*H*-indene (6):** White needle crystals; mp: 150–152 °C; 2.92 g, isolated yield 66%; ^1^H NMR (CDCl_3_, 300 MHz, ppm) δ_H_ 4.32–4.13 (m, 4H), 2.73–2.63 (m, 2H), 2.58–2.48 (m, 1H), 2.45–2.30 (m, 2H), 2.28–2.00 (m, 3H); ^13^C NMR (CDCl_3_, 75 MHz, ppm) δ_C_ 59.7 (CH), 53.6 (CH), 52.9 (CH), 52.5 (CH), 47.9 (CH), 41.1 (CH_2_), 38.5 (CH_2_), 37.0 (CH), 34.3 (CH_2_); FT IR (cm^−1^) *v* 2982, 2951, 2873, 2793, 1285, 1160, 1152, 1090, 1073, 1065, 893, 832, 758, 712, 690, 685; Anal. calcd for C_9_H_12_Br_4_ (439.80): C, 24.58; H, 2.75; Found: C, 24.70; H, 2.85.

***Rel*-(1S,2S,5S,6S)-1,2,5,6-tetrabromooctahydro-1*H*-indene (7):** Colorless oily liquid; 1.23 g, isolated yield 28%; ^1^H NMR (CDCl_3_, 300 MHz, ppm) δ_H_ 4.76 (dd, ^3^*J* = 8.5 Hz, ^3^*J* = 7.4 Hz, 1H), 4.55 (dd, ^3^*J* = 8.8 Hz, ^3^*J* = 7.9 Hz, 1H), 4.42 (dd, ^3^*J* = 9.1 Hz, ^3^*J* = 7.9 Hz, 1H), 4.24 (dd, ^3^*J* = 8.7 Hz, ^3^*J* = 7.4 Hz, 1H), 2.77–2.57 (m, 3H), 2.45–2.35 (m, 2H), 2.30–2.23 (m, 1H), 2.07–2.02 (m, 1H), 1.96–1.88 (m, 1H); ^13^C NMR (CDCl_3_, 75 MHz, ppm) δ_C_ 60.2 (CH), 53.0 (CH), 52.8 (CH), 50.3 (CH), 46.5 (CH), 40.5 (CH_2_), 33.3 (CH_2_), 33.0 (CH), 29.6 (CH_2_); FT IR (cm^−1^) *v* 2985, 2961, 2870, 2782, 1305, 1260, 1134, 1109, 1059, 1045, 898, 854, 743, 722, 695, 673; Anal. calcd for C_9_H_12_Br_4_ (439.80): C, 24.58; H, 2.75; Found: C, 24.70; H, 2.85.

### 2.5. Calculation method

The molecular structures of compounds **2**–**7** were drawn using GaussView 6.0.16 software [27]. Subsequently, quantum chemical calculations were performed to optimize the structures and obtain various properties using the Gaussian 09 AS64L-G09RevD.01 program at the B3LYP/6-31G(d) level of theory [28]. The B3LYP method combines hybrid density functional theory with a 6-31G(d) polarized basis set [29,30].

During the calculations, several properties were determined, including the total energy (E) and Gibbs free energy (G°) of the compounds, vibration frequencies, quantum chemical parameters, frontier molecular orbital contour diagrams, and MEP maps. These calculations provided valuable insights into the electronic structure and reactivity of the molecules.

The quantum chemical parameters were selected to compare the biological activities of the epoxide species. These parameters included the highest occupied molecular orbital energy (E_HOMO_), lowest unoccupied molecular orbital energy (E_LUMO_), energy gap (ΔE), hardness (η), softness (σ), electronegativity (χ), chemical potential (μ), electrophilic index (ω), nucleophilicity index (ɛ), electron acceptance power (ω^+^), and electron donation power (ω^−^). These parameters were determined using conceptual density functional theory and calculated using the appropriate equations from among Eqs. (1)–(9) [31–33].

Overall, these computational calculations and quantum chemical parameters provide valuable information about the structural, energetic, and electronic properties of compounds **2**–**7**, allowing for a better understanding of their potential biological activities and reactivity.


(1) $$I=-E_{HOMO}$$

(2) $$A=-E_{LUMO}$$

(3) $$\eta =\frac{1}{2}{\left[\frac{\partial^{2}E}{\partial N^{2}}\right]}_{\nu (r)}=\frac{I-A}{2}$$

(4) σ=1/η

(5) $$\mu =-\chi ={\left[\frac{\partial E}{\partial N}\right]}_{\nu (r)}=-\left(\frac{I+A}{2}\right)$$

(6) $$\omega =\chi^{2}/2\eta$$

(7) ɛ=1/ω

(8) $$\omega^{+}={\left(I+3A\right)}^{2}/(16(I-A))$$

(9) $$\omega^{-}={\left(3I+A\right)}^{2}/(16(I-A))$$

### 2.6. Docking procedure

The PDB files for compounds **2**–**7** and 5-FU were prepared based on their optimized structures. Molecular docking studies were conducted using DockingServer [34]. The geometry optimization of the recombinant drug candidate compounds and proteins was performed using the MMFF94 method within DockingServer [35]. The Gasteiger method was utilized for load calculations, and all calculations were carried out at pH 7.0.

For the docking simulations, the grid maps were set to dimensions of 90 × 90 × 90 Å (x, y, and z), and the Solis and Wets local search method Lamarckian genetic algorithm processor was employed. The docking parameters included a population size of 150, a translation step of 0.2 Å, and quaternion and torsion steps of 5 Å. These settings allowed for the exploration of the appropriate region of the target protein for the molecules under investigation.

The results of the docking simulations provided various energy terms, including the binding energy (BE), intermolecular energy (IE), van der Waals energy, H-bond energy, and van der Waals + hydrogen bond + desolvation energy (WHDE), as well as the estimated inhibition constant (K_i_) and interaction surface (IS) between the drug candidate molecules and target proteins.

By analyzing the docking results, valuable insights could be gained into the binding affinities, intermolecular interactions, and potential inhibitory activities of the drug candidates with the target proteins.

### 2.7. X-ray crystallography

For the crystal structure determination of dibromodiacetate **3**, a single crystal was used for data collection on a Rigaku R-AXIS RAPID-S diffractometer (Rigaku Corporation, Tokyo, Japan) equipped with a two-dimensional area IP detector. Graphite-monochromated Mo-Kα radiation with a wavelength (λ) of 0.71073 Å was employed and data collection was performed using the oscillation scan technique with a width (Δ*w*) of 5° for one image.

The lattice parameters were determined by applying least-squares methods to all reflections with *F^2^* > 2*σ*(*F^2^*). Data integration, correction for Lorentz and polarization effects, and cell refinement were carried out using CrystalClear software [36].

The crystal structures were solved using SHELXS-97, employing direct methods to locate most of the heaviest atoms. The remaining nonhydrogen atoms were located by utilizing Fourier maps generated from successive full-matrix least-squares refinement cycles on *F^2^* using SHELXL-97 [37].

All nonhydrogen atoms were refined with anisotropic displacement parameters. Hydrogen atoms attached to carbon atoms were positioned at their geometric positions using appropriate HFIX instructions in SHELXL. The final difference Fourier maps revealed no peaks of chemical significance, indicating a good fit between the model and the experimental data.

Crystal data for **3**: C_13_H_18_O_4_Br_2_, crystal system, space group: monoclinic, P2_1_/n; (no: 14); unit cell dimensions: *a* = 13.8363(4), *b* = 7.7344(3), *c* = 14.7282(5) Å, *α* = 90, *β* = 97.945(4), *γ* = 90°; volume; 1557.63(9) Å^3^, Z = 4; calculated density: 1.697 g/cm^3^; absorption coefficient: 5.212 mm^−1^; *F*(000): 792; *θ* range for data collection 2.8° to 30.5°; refinement method: full-matrix least-square on *F*^2^; data/parameters: 2904/175; goodness-of-fit on *F*^2^: 1.350; final *R*-indices [*I* > 2s(*I*)]: *R*_1_ = 0.089, w*R*_2_ = 0.222; largest diff. peak and hole: 0.798 and −0.672 e Å^−3^.

## 3. Results and discussion

### 3.1. Synthesis and structural characterization

**Figure 1:**
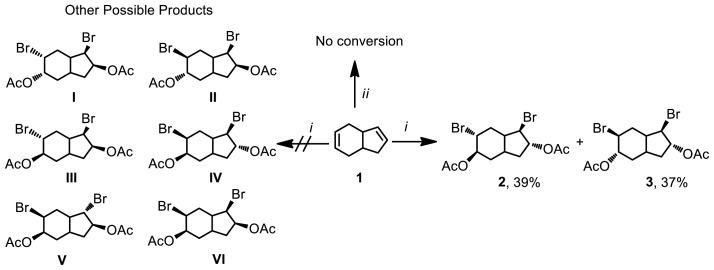
Selective synthesis of dibromoacetate (**2, 3**) and other possible products. *i*. NBS, LiClO_4_, CH_3_COOH, 48 h, rt; *ii*. NBS, CH_3_COOH, 60 h, rt.

**Figure 2:**
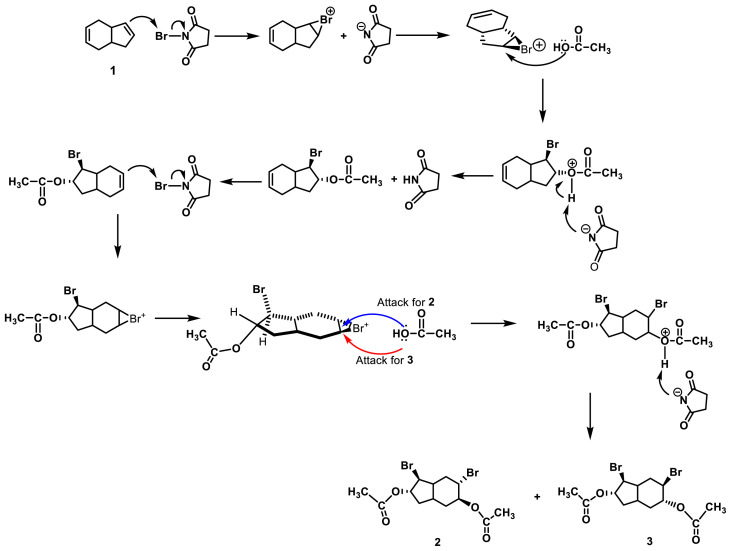
Supposed mechanistic approach to synthesis of dibromoacetates **2** and **3**.

In this study, a novel approach for the synthesis of diepoxides is proposed. Tetrahydroindene 1 undergoes bromination with NBS in the presence of LiClO_4_ as a Lewis acid catalyst in acetic acid, resulting in the formation of a mixture of dibromodiacetate derivatives 2 and 3 at room temperature. The separation of the mixture of dibromodiacetates 2 and 3 can be achieved through column chromatography, providing yields of 39% and 37%, respectively (Figures 1 and 2).

The use of LiClO_4_ as a Lewis acid catalyst in the bromoacetate synthesis reaction offers several advantages over catalyst-free trials [26]. It enhances the reaction rate, making it faster. Furthermore, the use of LiClO_4_ as a catalyst creates a more selective reaction environment, resulting in the formation of more selective products compared to the possible bromoacetate products (Figure 1). This increased selectivity is beneficial in achieving the desired compounds and can be attributed to the influence of LiClO_4_ on the reaction mechanism due to improved polarization [24,25].

**Figure 3:**
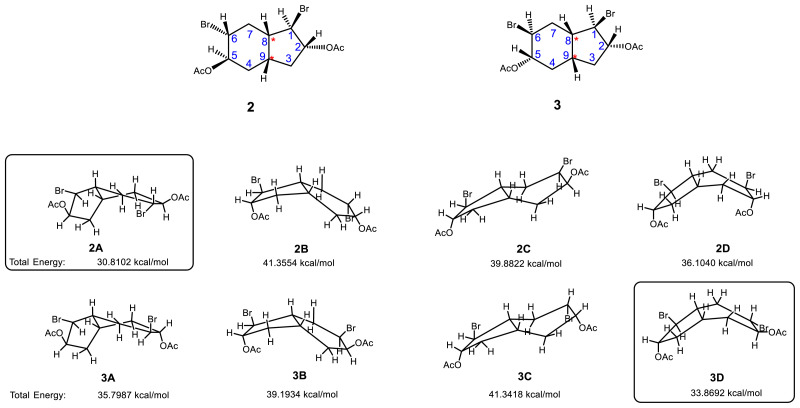
Conformations of stereoisomeric dibromodiacetate tetrahydroindene compounds (**2A**–**3D**) and their total energies (kcal/mol), calculated by ChemDraw 3D (Chem Office 2015 version). Energies were calculated relative to 2A and 3D and to the most stable conformer for each isomer. *: The bridgehead stereocenters were specified as the *R* configuration.

The possible conformations of stereoisomeric dibromodiacetate tetrahydroindene compounds (2A–3D) were drawn and their total energies (kcal/mol) were calculated to identify the most stable conformer for each stereoisomer (2A and 3D) (Figure 3). Furthermore, the possible conformations of stereoisomeric tetrahydroindene diepoxides 4 and 5 were confirmed with the literature [11].

**Figure 4:**
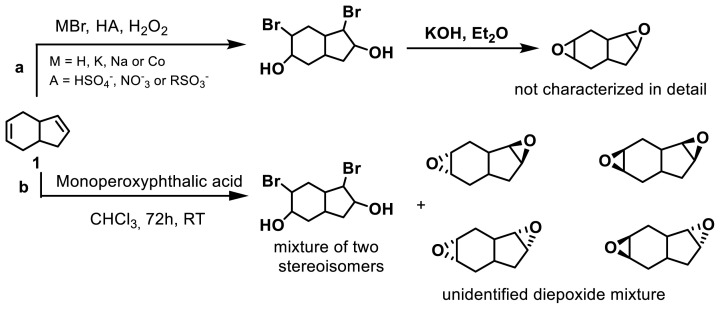
The synthetic approach to diepoxides by the procedures of Alimardanov et al. [38] (**a**) and Okovytyy et al. [11] (**b**).

**Figure 5:**
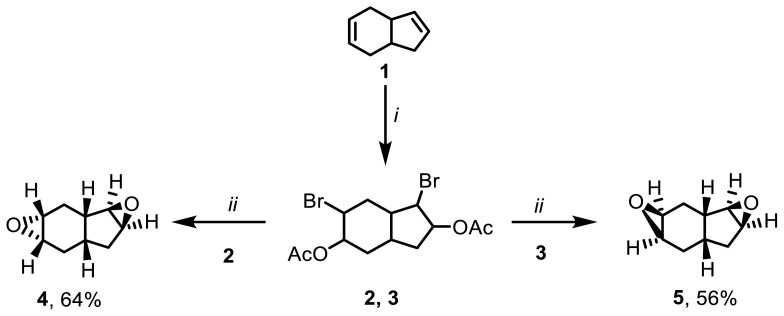
Our approach to the synthesis of diepoxides (**4**, **5**) *i*. NBS, LiClO_4_, CH_3_COOH, 48 h, rt; *ii*. NaOH, CH_3_OH, 44 h, rt.

To obtain the epoxy derivatives, the bromoacetate indene mixture was initially treated with NaOH in methanol, resulting in a mixture of diepoxides 4 and 5. Subsequently, the isomers were purified using column chromatography, yielding diepoxide 4 and diepoxide 5 in yields of 41% and 23%, respectively. Additionally, after purifying dibromodiacetate compounds 2 and 3 through column chromatography, each isomer was individually subjected to epoxidation under the same conditions. The epoxidation processes of dibromodiacetate compounds 2 and 3 selectively produced diepoxides 4 and 5 as the sole products, with moderate yields of 64% and 56%, respectively. In the literature, diepoxides have been synthesized by treating tetrahydroindene with monoperoxyphthalic acid, resulting in the formation of four diepoxy indene stereoisomers with different orientations [11]. Additionally, in another study by Alimardanov et al., halohydrins were synthesized using hydrogen halides and hydrogen peroxide. It was reported that the formed halohydrins can be further converted into three epoxide derivatives by reaction with an inorganic base in an organic solvent [38] (Figure 4). However, the detailed structural characterization of the diepoxide products obtained in both of those previous studies was not performed using NMR analysis. In our study, dibromodiacetate stereoisomers 2 and 3 selectively yielded diepoxides 4 and **5** with endo-exo and exo-exo orientations, respectively (Figure 5).

This study proposes an alternative method for the facile conversion of brominated indenes bearing acetate to epoxides. This method provides a route to access epoxides in a selective and specific manner as an alternative to the synthesis of many chemicals.

**Figure 6:**
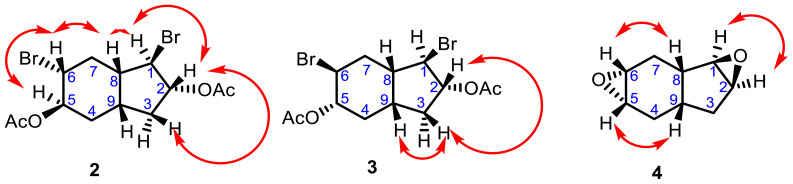
Some COSY correlations of compounds **2**, **3**, and **4**.

**Figure 7:**
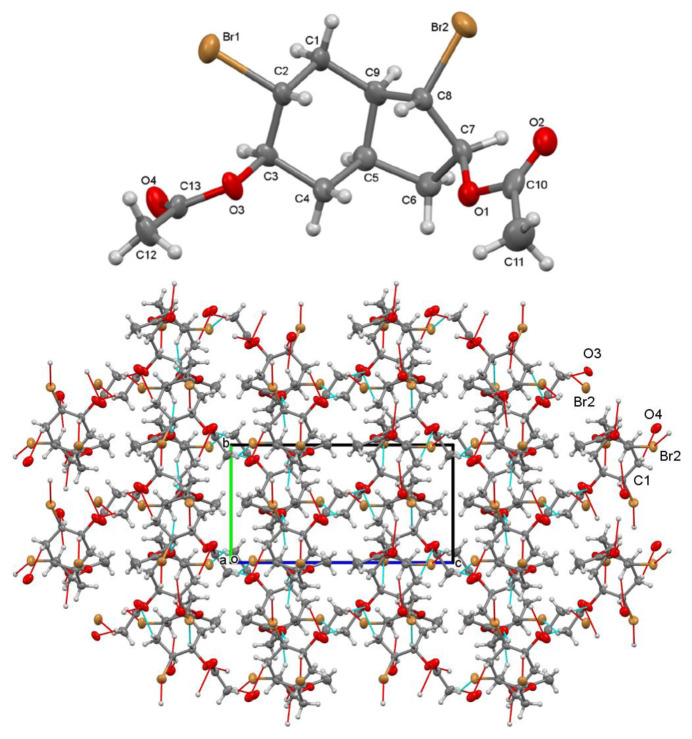
(Top) X-ray structure of molecule **3**. Thermal ellipsoids are drawn at the 40% probability level. (Bottom) Stacking motif with the unit cell viewed down along the *a*-axis. Dashed red lines indicate interactions less than the sum of the vdW radii.

**Figure 8:**
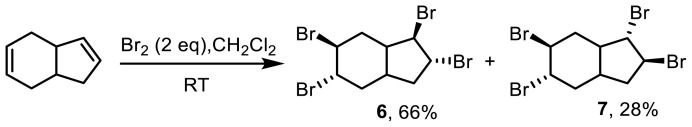
Synthesis of tetrabromoindene (**6** and **7**).

The structures of novel dibromodiacetate stereoisomers 2 and 3 were characterized by ^1^H and ^13^C NMR, 2D NMR (HETCOR, COSY), FT IR, and elemental analysis. The NMR spectra are available in the Supporting Information (Figures 1S–8S). The ^1^H NMR spectra of 2 and 3 consist of ten aliphatic signals each. The resonance for six methyl protons at approximately 1.96 and 2.09 ppm indicated that two acetate groups bonded to the indene rings of 2 and 3. In the APT spectra of **2** and **3**, two quaternary C signals at approximately δ_C_ 170.0 ppm and two methyl C signals at δ_C_ 21.3 and 21.2 ppm verified the presence of acetate groups on the indene rings (Table 1). Furthermore, in the FT IR spectra of 2 and 3, the characteristic C=O bond stretching was observed at 1737 and 1732 cm^−1^, respectively. In the HETCOR spectrum of 2, the multiplet signals of H_2_ and H_5_ (δ_H_ 5.15–5.09 and 4.88–4.82 ppm, respectively) correlated with methine carbon signals (δ_C_ 82.0 and 72.8 ppm, respectively). These findings showed the acetate groups bound to those positions. In the COSY experiment performed for **2**, the multiplet signal at δ_H_ 5.15–5.09 ppm correlated with the multiplet signals at δ_H_ 4.13–4.04 and 2.51–2.32 ppm, indicating that the signals at δ_H_ 4.13–4.04 and 2.51–2.32 ppm belonged to the C_1_ and C_3_ positions (Figure 6 and Table 1). The APT and COSY spectral experiments for **3** gave correlations similar to those for **2** (Figure 6; Table 1).

The structures of diepoxide stereoisomers 4 and 5 were identified by ^1^H and ^13^C NMR, 2D NMR (HETCOR, COSY) (Supporting Information Figures 9S–18S), FT IR, and elemental analysis. In the ^1^H NMR spectra of 4 and 5, proton signals were observed more upfield compared to starting compounds 2 and 3 due to the carbonyl groups and bromine atoms leaving the indene rings. Moreover, the disappearance of two quaternary C signals at approximately δ_C_ 170.0 ppm and two methyl C signals at δ_C_ 21.3 and 21.2 ppm in the ^13^C NMR spectra of 4 and 5 and the characteristic C=O bond stretching at 1737 and 1732 cm^−1^ in the FT IR spectra confirmed the structure of diepoxides 4 and 5. In addition, the ^13^C NMR and APT spectra of 4 and 5, consisting of 6 methine and 3 methylene signals, were also in good agreement with the proposed structures. The reported spectral data [11] confirmed our experimental data. Atom numbers are labeled according to the compounds in Figure 6.

The conformation of 1,6-dibromoctahydro-1H-indene-2,5-diyl diacetate (**3**) was determined through X-ray diffraction analysis, as shown in Figure 7. The crystal structure of molecule **3** belongs to the monoclinic space group P21/n, with four molecules present in the unit cell (Table 2). The cyclohexane unit adopts a chair conformation, which is energetically favorable. The C-C (cyclohexane) bond lengths range from 1.512(3) to 1.530(3) Å, indicating a single bond character. The bond distances for C_2_-Br_1_ and C_8_-Br_2_ are 1.972(3) and 1.945(3) Å, respectively.

The racemic bicyclic structure of compound **3** contains six asymmetric carbon atoms and the stereogenic centers are as follows: C_2_(*RS*), C_3_(*RS*), C_5_(*RS*), C_7_(*SR*), C_8_(*SR*), and C_9_(*RS*). In the solid state, compound **3** is stabilized through weak intermolecular C_12_-H···O_3_ hydrogen bonds, where the donor-acceptor distance is *D*···*A* = 3.484(3) Å. This interaction leads to the formation of a dimeric structure with each enantiomer forming a distinct dimer. Van der Waals (vdW) interactions also contribute to the overall stability of the structure (Figure 7).

Tetrahydro-1H-indene was also treated with molecular bromine (2 equivalents) in CH_2_Cl_2_ at ambient temperature, resulting in the formation of a mixture of two tetrabromide isomers, namely 6 and 7 (Figure 8). Novel tetrabromo tetrahydro-1H-indene isomers 6 and 7 were successfully isolated as the sole products through column chromatography with yields of 66% and 28%, respectively. The structures of tetrabromides 6 and 7 were characterized by spectroscopic methods including ^1^H and ^13^C NMR, FTIR, and elemental analysis. The spectral data were confirmed by the literature [39].

### 3.2. Optimized structure

**Figure 9:**
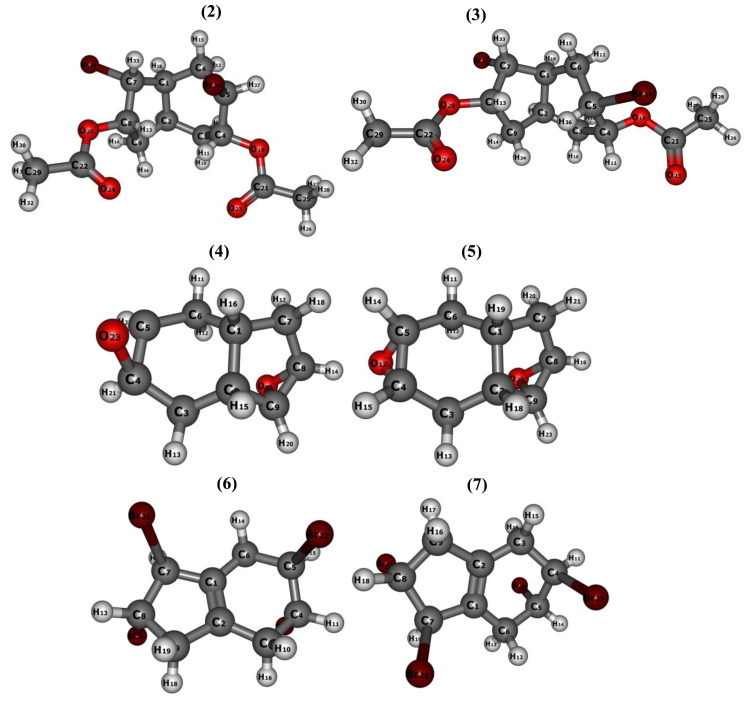
Optimized structures of **2**–**7**.

The ground-state optimization structures of compounds **2**–**7** were obtained using the B3LYP/6-31G(d) level of theory and the resulting structures are depicted in Figure 9.

Upon evaluating the optimized structures of the investigated compounds, it was observed that there were no discernible differences in the geometric structures among the stereoisomers. This conclusion was reached by comparing various geometric parameters such as bond lengths and bond angles. For instance, the C_6_-C_1_-C_7_ bond angle was determined to be 115.0° for the combination of compounds **2** and **3**, while the C_3_-C_2_-C_9_ bond angle in both compounds **2** and **3** was found to be 114.0°. The bond lengths of C_5_-Br and C_7_-Br in these compounds were calculated to be 2.00 Å. In the case of compound **4**, the C_5_-O_23_-C_4_ bond angle is 60.9° and the C_8_-O_19_-C_9_ bond angle is 61.0°. This slight difference may arise from the formation of a cyclo-structure within the six-ring and the five-ring, respectively. A similar situation holds for compound **5**. In compound **6**, the bond lengths of C_4_-Br_22_, C_5_-Br_23_, C_7_-Br_21_, and C_8_-Br_20_ are approximately 2.0 Å. Moreover, the C_3_-C_2_-C_9_ bond angle was measured as 124°. The same observations are valid for compound **7**. Since compounds **2**–**7** lack aromaticity, their geometries are nonplanar.

### 3.3. Stability of stereoisomers

The stability of isomers with the same number of electrons can be predicted using various thermodynamic parameters, including total energy (E_Total_), enthalpy (H), and Gibbs free energy (G) [40,41]. In this study, the stability of the investigated stereoisomers was assessed by comparing compounds 2 and 3, 4 and 5, and 6 and 7 using these thermodynamic parameters, and the calculated values are presented in Table 3.

E_Total_, H, and G energy values for compounds with an equal number of electrons tend to yield similar results. However, these values can still be utilized to explain the relative stability of the isomers. The stereoisomer with the lowest energy corresponds to the most stable configuration. Based on the calculated E_Total_, H, and G values, the stability order of the stereoisomers was determined as follows: 3 > 2, 4 > 5, and 7 > 6.

### 3.4. Contour diagrams and MEP maps

Contour diagrams and MEP maps provide valuable insights into the reactive sites of epoxide derivatives. These diagrams and maps are generated by calculating the electronic charges of each atom in a molecule, allowing the identification of electron donor and acceptor regions based on frontier molecular orbitals. By visualizing the electron localization regions, regions of both high and low electron density can be easily observed from the MEP maps.

**Figure 10:**
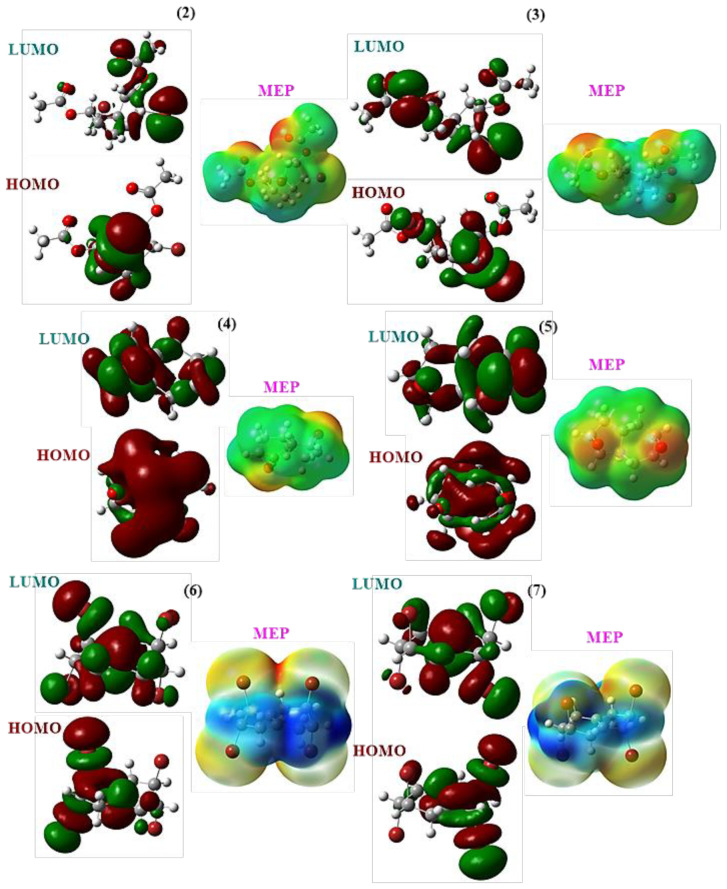
Frontier molecular orbitals and MEP maps of **2**–**7**.

Figure 10 displays the shapes of the highest occupied molecular orbital (HOMO) and lowest unoccupied molecular orbital (LUMO) for compounds 2–7. In the epoxide group ring of compounds 4 and 5, as well as the bromine atoms in structures 2 and 3, π-molecular orbitals can be observed. The LUMO indicates the presence of π*-molecular orbitals on the epoxide group ring. Similarly, compounds 6 and 7 exhibit similar HOMO and LUMO results. Therefore, it can be inferred that the HOMO → LUMO transition corresponds to the π-π* transition. The MEP maps highlight the region with the highest electron density, which is predominantly located on the oxygen atoms. Consequently, it can be predicted that these oxygen atoms would engage in interactions with nucleophilic species approaching the investigated molecules.

### 3.5. Molecular activity with some quantum chemical parameters

In recent years, the use of quantum chemical parameters has emerged as a promising approach for predicting molecular reactivity and distinguishing the molecular properties of compounds with similar geometric structures [42]. These parameters allow us to obtain information about isomers and spatial differences between atomically distinct components. Table 4 presents several quantum chemical parameters, including the highest occupied molecular orbital energy (E_HOMO_), lowest unoccupied molecular orbital energy (E_LUMO_), energy gap (ΔE), hardness (η), softness (σ), electronegativity (χ), chemical potential (μ), electrophilic index (ω), nucleophilicity index (ɛ), electron acceptance power (ω^+^), and electron donation power (ω^−^), as calculated using Eqs. (1)–(9). Additionally, the anticancer drug 5-FU was included as a reference substance for the evaluation of anticancer activity.

Comparing the molecular descriptors of compounds 2 and 3, 4 and 5, 6 and 7, and 5-FU, it can be observed that some descriptors are numerically smaller or larger. Based on the molecular structure descriptors provided in Table 4, it can be predicted that an increase in anticancer activity is associated with an increase in E_HOMO_, I, σ, μ, ɛ, and ω^−^ and a decrease in E_LUMO_, A, ΔE, η, χ, ω, and ω^+^. Consequently, the molecular anticancer activities can be comparatively ranked as follows: 2 > 3 > 7 > 6 > 5 > 4 > 5-FU.

### 3.6. Molecular docking

Molecular docking is a widely used technique in the investigation of the molecular-level activities of biologically important compounds. It provides insights into the interactions between drug candidate molecules and biomolecules such as DNA, RNA, and proteins.

Docking studies of epoxides, which have not yet been studied at the molecular level, and examinations of the interaction sites and interaction types with amino acid residues in the protein structure can provide extremely useful information to drug designers. In this study, it was aimed to investigate the anticancer properties of compounds **2**–**7** against ovarian (A2780), breast (MCF-7), colon (HT-29), and lung (H1299) cancer cell lines. The molecular docking results of the investigated compounds were compared with those of antitumor standard 5-FU. Target proteins were selected from the Protein Data Bank according to the cell line they represented. PDB ID: 1SA1 (for A2780) is a stathmin-like domain complex that is tubulin-podophyllotoxin. Microtubules, the cytoskeletal polymers of tubulin, are controlled by a large number of compounds and proteins, such as proteins of the stathmin family. This particular protein represents the structure of tubulin in complex with the stathmin-like domain (SLD) [43]. It has been demonstrated that tubulin is a multifunctional protein that has a key role in the pathobiology and aggression of human lung cancer by affecting tumor incidence and progression [44]. The 10-formyltetrahydrofolate structure of human 5,10-methenyltetrahydrofolate synthetase (MTHFS) is the PDB ID: 3HY3 target protein for MCF-7. MTHFS has been determined to regulate the flow of carbon through the one-carbon metabolic network that provides the components necessary for cells to grow and reproduce. Inhibition of MTHFS in human MCF-7 breast cancer cells has been shown to stop the growth of the cells [45]. PDB ID: 6ECZ represents selectively tumor-associated carbonic anhydrase isoforms containing bioreductive 4-hydroxy-3-nitro-5-ureido-benzenesulfonamides. Human carbonic anhydrases carry cellular properties characteristic of many tumors. The 6ECZ protein sequence provided the representation of the antitumor CAIs of HT-29, MDA-MB-231, and PC-3 human cancer cell lines in both normoxic and hypoxic conditions [46]. The PDB ID: 6FL5 target protein, consisting of three SHMT1 mutants that differentiate the oligomeric state of the enzyme from its catalytic activity, represents lung cancer cells (A549 and H1299) [47]. It was docked between compounds 2–7 and target proteins 1SA1, 3HY3, 6ECZ, and 6FL5, and some docking parameters such as energy of binding (BE), vdW + Hbond + desolved energy (WHDE), estimated inhibition constant (K_i_), and interaction surface (IS) are given in Table 5.

Based on the energy values presented in Table 5, it can said that the most inhibited target protein is PDB ID: 3HY3, which is targeted by compounds 2–7. On the other hand, the studied compounds exhibited the least inhibitory activity against the 6FL5 target protein. Specifically, compound 2 showed the highest anticancer effect against the MCF-7 cell line while compound 4 exhibited the lowest activity.

**Figure 11:**
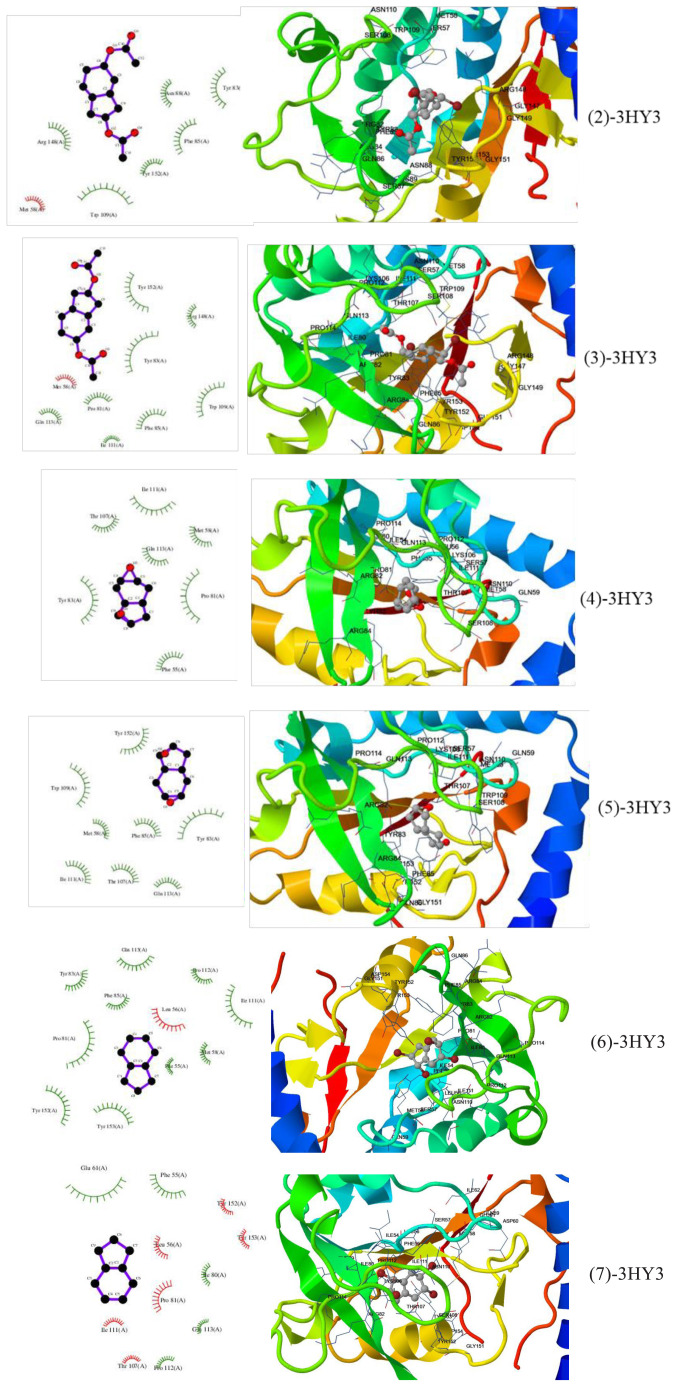
The receptor–ligand structure between the highest biological activity of **2–7** and the 3HY3 target protein.

The estimated binding energy between the 3HY3 target protein and compound 2 is −7.92 kcal/mol, while the energy produced by secondary chemical interactions is −6.03 kcal/mol. The types of interactions between the complexes and target proteins are described in Table 6. Figure 11 illustrates the receptor–ligand structure between the 3HY3 target protein and the compound with the highest biological activity among compounds 2–7.

The BE and WHDE values provide insights into the interaction strength between the diepoxybicyclo compounds and the target proteins (Table 6). Negative values indicate an increase in inhibitory power, reflecting stronger interactions between the compounds and the target proteins associated with the cancer cell lines.

Furthermore, it is noteworthy that all studied compounds exhibited higher potency than reference compound 5-FU against the cancer cell lines, except for the lung cancer cell lines. The relative anticancer potentials among the studied target proteins and compounds can be summarized as follows: 2 > 7 > 3 > 6 > 5 > 4 > 5-FU against the A2780 and MCF-7 cell lines and 6 > 7 > 2 > 5 > 4 > 3 > 5-FU against the HT-29 and H1299 cell lines.

It is important to note that further experimental studies are required to validate and confirm the actual anticancer activities of the investigated compounds.

The first order presented above is in good agreement with the activity order suggested by the quantum chemical parameters. In addition, this order is inversely proportional to the structural stability of the studied diepoxybicyclo compounds. This may lead to unstable compounds interacting better with proteins to stabilize them.

K_i_ is a measure of whether a drug active ingredient is able to inhibit the cell line under investigation, as well as the ligand’s protein binding affinity. If it is smaller, a smaller amount of the drug is required to inhibit enzyme activity [48]. These results can be seen in Table 5, with the compounds showing inhibitory activity on the micromolar (μM) level.

Tables 5 and 6 detail the types of secondary chemical interactions between the compounds and amino acid residues of the target proteins. The types of interactions between target proteins and the compounds studied are generally polar and hydrophobic interactions. In addition to these interactions, 2, 3, 6, and 7 exhibit halogen bonds and 4 and 5 show π-π interactions. These interactions provide information on the molecular-scale chemical relationships between the drug candidates and the studied cancer cell lines.

## 4. Conclusion

In this study, the synthesis of diepoxy bicyclic nonanes 4 and 5 using the proposed method proved to be a convenient and efficient approach, allowing for selective formation of endo-exo and exo-exo orientations. This synthetic route offers a short and practical method for obtaining specific and selective diepoxide compounds. Additionally, the direct bromination of tetrahydro-1H-indene resulted in the formation of tetrabromide isomers 6 and 7.

The stability of the stereoisomers was determined as 2 > 3, 4 > 5, and 7 > 6. Analysis of frontier molecular orbitals revealed the exchange between π-molecular orbitals and π*-molecular orbitals in the epoxide group ring. MEP maps highlighted the region with the highest electron density on the oxygen atoms. Quantum chemical parameters such as E_HOMO_, E_LUMO_, ΔE, η, σ, χ, μ, ω, ɛ, ω^+^, and ω^−^ indicated the relative biological activities of the compounds, with compound 2 exhibiting the highest activity among the tested species, followed by 3, 5, 4, 7, 6, and 5-FU.

Molecular docking studies against ovarian (A2780), breast (MCF-7), colon (HT-29), and lung (H1299) cancer cell lines revealed the anticancer properties of the compounds. Compound 2 exhibited the most effective anticancer activity against MCF-7 cells. The overall order of anticancer activities across the studied cell lines was 2 > 7 > 3 > 6 > 5 > 4 > 5-FU. The results of quantum chemical parameters and docking studies were found to be consistent with each other. It is worth noting that the stability order of the isomers and the order of anticancer activity exhibited opposite trends, suggesting that certain compounds may form more efficient receptor–ligand complexes with biological systems, enhancing their stability and interaction.

Further experimental studies are necessary to validate the observed anticancer activities and confirm the potential of the investigated compounds as drug candidates.

## Supplementary Information (SI)

The Supplementary Information contains all the ^1^H NMR, ^13^C NMR, and IR spectra of the synthesized compounds.

CCDC-2169297 contains the supplementary crystallographic data for structure **3**. These data are provided free of charge via the joint CCDC/FIZ Karlsruhe deposition service at www.ccdc.cam.ac.uk/structures .

Figure 1S ^1^H NMR spectrum of dibromodiacetate **2** (300 MHz, in CDCl_3_).

Figure 2S ^13^C NMR spectrum of dibromodiacetate **2** (75 MHz, in CDCl_3_).

Figure 3S APT spectrum of dibromodiacetate **2**.

Figure 4S COSY spectrum of dibromodiacetate **2**.

Figure 5S HETCOR spectrum of dibromodiacetate **2**.

Figure 6S ^1^H NMR spectrum of dibromodiacetate **3** (300 MHz, in CDCl_3_).

Figure 7S APT spectrum of product dibromodiacetate **3**.

Figure 8S COSY spectrum of product dibromodiacetate **3**.

Figure 9S ^1^H NMR spectrum of diepoxide **4**.

Figure 10S ^13^C NMR spectrum of diepoxide **4** (75 MHz, in CDCl_3_).

Figure 11S APT spectrum of diepoxide **4**.

Figure 12S COSY spectrum of diepoxide **4**.

Figure 13S HETCOR spectrum of product diepoxide **4**.

Figure 14S ^1^H NMR spectrum of product diepoxide **5** (300 MHz, in CDCl_3_).

Figure 15S APT spectrum of diepoxide **5**.

Figure 16S COSY spectrum of diepoxide **5**.

Figure 17S ^1^H NMR and ^13^C NMR spectra of tetrabromide **6** (300/75 MHz, in CDCl_3_).

Figure 18S ^1^H NMR and ^13^C NMR spectra of tetrabromide **7** (300/75 MHz, in CDCl_3_).

## Figures and Tables

**Table 1:** ^1^H NMR, ^13^C NMR, APT, and COSY experimental data of compounds **2**, **3**, and **4** (300 MHz, CDCl_3_).

2				3			4		
C/H	δ_H_ ppm	δ_C_ ppm (APT)	COSY	δ_H_ ppm	δ_C_ ppm (APT)	COSY	δ_H_ ppm	δ_C_ ppm (APT)	COSY
1	4.13–4.04	48.6 (CH)	H_2_, H_8_	4.13–3.99	48.3 (CH)	H_2_, H_8_	3.44–3.42	59.2 (CH)	H_2_, H_8_
2	5.15–5.09	82.0 (CH)	H_9_, H_3a_, H_1_	5.29–5.23	82.2 (CH)	H_9_, H_3a_, H_1_	3.11–3.07	51.4 (CH)	H_9_, H_3b_
3/a	2.51–2.32	33.9 (CH_2_)	H_2_	2.53–2.44	37.1 (CH_2_)	H_9_, H_2_, H_1_	2.03–1.89	33.8 (CH_2_)	H_4a_
3/b	2.14–2.00	33.9 (CH_2_)	H_1_	1.74–1.35	37.1 (CH_2_)		1.68–1.55	33.8 (CH_2_)	H_2_, H_4a_, H_9_
4/a	2.14–2.00	27.2 (CH_2_)	H_1_, H_5_	2.33–2.20	33.7 (CH_2_)		2.03–1.89	32.1 (CH_2_)	H_3b_, H_6_
4/b	1.72–1.62	27.2 (CH_2_)	H_5_, H_8_	2.19–2.12	33.7 (CH_2_)	H_5_, H_9_	1.27–1.15	32.1 (CH_2_)	H_6_, H_3a_
5	4.88–4.82	72.8 (CH)	H_4b_, H_7a_, H_6_	4.90–4.82	75.2 (CH)	H_4b_, H_9_, H_6_	3.29–3.27	62.2 (CH)	H_9_, H_7b_
6	4.13–4.04	56.4 (CH)	H_5_, H_8_	4.13–3.99	53.1 (CH)	H_5_, H_7a_	3.03–2.98	51.2 (CH)	H_4b_, H_8_
7/a	2.23–2.15	35.2 (CH_2_)	H_5_	2.67–2.60	34.8 (CH_2_)	H_6_, H_8_	1.68–1.55	24.9 (CH_2_)	H_8_, H_9_
7/b	2.51–2.32	35.2 (CH_2_)	H_9_	2.33–2.20	34.8 (CH_2_)		2.28–2.15	24.9 (CH_2_)	H_6_
8	2.23–2.15	33.7 (CH)	H_6_, H_4b_	2.19–2.12	35.2 (CH)	H_7a_, H_1_	2.03–1.89	31.6 (CH)	H_6_, H_4b_, H_7a_
9	1.38–1.29	45.3 (CH)	H_2_	1.74–1.35	47.6 (CH)	H_4b_, H_3a_	2.28–2.15	36.6 (CH)	H_3b_
10	-	170.0 (C)		-	170.3 (C)				
11	-	169.8 (C)		-	170.2 (C)				
12	1.96	21.2 (CH_3_)		21.2	21.2 (CH_3_)				
13	1.94	21.3 (CH_3_)		21.3	21.3 (CH_3_)				

**Table 2:** Crystal data and structure refinement parameters for **3**.

CCDC deposition number	2169297
Empirical formula	C_13_H_18_O_4_Br_2_
*M*_r_	398.09
Crystal system, space group	Monoclinic, P2_1_/n
Temperature (K)	296
*a*, *b*, *c* (Å)	*a* 13.8363(4), 7.7344(3), 14.7282(5)
*α* (°)	90
*β* (°)	97.945(4)
*γ* (°)	90
*V* (Å^3^)	1557.63(9)
*Z*	4
*F*(000)	792
*D_x_* (g/cm^3^)	1.697
Radiation type	MoK*α*
l (Å)	0.71073
*θ* range (°) for cell measurement	2.8°–30.5°
*μ* (mm^−1^)	5.212
Crystal shape	Prism
Color	Colorless
Crystal size (mm)	0.04 × 0.07 × 0.16
Refinement on	*F* ^2^
Final *R*-indices [*I* > 2s(*I*)]: *R*_1_, w*R*_2_	0.089, 0.222
Goodness-of-fit on *F*^2^	1.350
Data/parameters	2904/175
*Δρ*_max_, *Δρ*_min_ (e Å^−3^)	0.798, −0.672

**Table 3:** Calculated thermodynamic parameters (atomic units) for stereoisomers 2 and 3, 4 and 5, and 6 and 7.

Compounds	Total energy	Enthalpy	Gibbs free energy
**2**	−5950.562151	−5950.240203	−5950.321966
**3**	−5950.569165	−5950.247327	−5950.322470
**4**	−500.5518140	−500.3440440	−500.3870510
**5**	−500.5465370	−500.3390670	−500.3817450
**6**	−10635.821871	−10635.635644	−10635.695415
**7**	−10635.821979	−10635.635756	−10635.695408

**Table 4:** The calculated quantum chemical parameters for **2**–**7**.

Compound	2	3	4	5	6	7	5-FU
E_HOMO_ (eV)	−6.3496	−6.5411	−7.0021	−6.9474	−6.8677	−6.8848	−7.0565
E_LUMO_ (eV)	−2.6605	−2.5628	−2.3002	−2.4058	−1.2972	−1.3432	−1.9192
ΔE (eV)	3.6891	3.9783	4.7019	4.5416	5.5705	5.5416	5.1373
η (eV)	1.8445	1.9892	2.3509	2.2708	2.7852	2.7708	2.5686
σ (eV^−1^)	0.5421	0.5027	0.4254	0.4404	0.3590	0.3609	0.3893
χ (eV)	4.5050	4.5520	4.6511	4.6766	4.0824	4.1140	4.4879
μ (eV^−1^)	−4.5050	−4.5520	−4.6511	−4. 6766	−4.0824	−4.1140	−4.4879
ω	5.5014	5.2083	4.6009	4.8156	2.9919	3.0241	3.9206
ɛ	0.1818	0.1920	0.2173	0.2077	0.3342	0.3274	0.2551
ω^+^	3.4795	3.1810	2.5692	2.7611	1.2988	1.3435	1.9977
ω^−^	7.9845	7.7329	7.2204	7.4377	5.3812	5.4575	6.4856

**Table 5:** Calculated docking results between compounds **2**–**7** and target proteins.

Cell line (target protein)	Compounds	BE (kcal/mol)	WHDE (kcal/mol)	K_i_ (μM)	IS
A2780 (1SA1)	**2**	−6.24	−7.27	26.56	875.894
	**3**	−5.97	−6.97	42.02	815.102
	**4**	−4.83	−4.62	286.45	551.444
	**5**	−4.88	−4.67	265.21	554.602
	**6**	−5.62	−5.60	75.98	569.845
	**7**	−6.07	−6.04	35.47	613.275
	5-FU	−3.54	−3.82	274.32	671.532
MCF-7 (3HY3)	**2**	−7.92	−6.03	249.24	654.859
	**3**	−7.18	−8.31	5.50	674.811
	**4**	−5.27	−5.23	115.96	449.561
	**5**	−5.30	−5.26	131.23	491.619
	**6**	−6.90	−6.91	8.73	454.897
	**7**	−7.17	−7.21	5.56	439.629
	5-FU	−4.65	−4.71	201.69	458.582
HT-29 (6ECZ)	**2**	−4.85	−5.98	277.13	607.332
	**3**	−4.75	−5.90	331.75	613.042
	**4**	−4.61	−4.57	421.19	416.654
	**5**	−4.68	−4.66	374.12	419.579
	**6**	−5.84	−5.85	52.45	395.507
	**7**	−5.76	−5.77	59.67	417.965
	5-FU	−3.96	−4.02	302.52	412.547
H1299 (6FL5)	**2**	−4.41	−5.58	580.00	498.893
	**3**	−3.14	−4.27	490.00	502.555
	**4**	−3.93	−3.91	1320.0	357.051
	**5**	−3.98	−3.94	731.50	349.050
	**6**	−5.24	−5.22	145.30	344.819
	**7**	−5.12	−5.13	177.43	343.446
	5-FU	−3.68	−3.76	774.22	332.063

**Table 6:** Interaction types of compounds **2**–**7** and 1SA1, 3HY3, 6ECZ, and 6FL5 target proteins in docking.

Interactions	H bond	Polar	Hydrophobic	Halogen bonds	π-π
**2**-1SA1	-	ASN101, THR145 THR179, GLU183	ALA12, ALA99	SER140, GLY143 THR145, TYR224	
**3**-1SA1	THR179	GLN11, GLU183		ASP69, THR145	
**4**-1SA1	SER140	THR145	ALA99	-	
**5**-1SA1	SER140	-	ALA99	-	
**6**-1SA1	-	-	ALA99	GLN11, ALA99, SER140, GLY143	
**7**-1SA1	-	-	ALA12, ILE171 PRO173, TYR224	GLY142, ILE171 VAL177, ASN206	
5-FU-1SA1		GLU183, THR179			
**2**-3HY3	-	TYR83, TYR152	MET58, TYR83 PHE85, TRP109 TYR152	TYR152	
**3**-3HY3		TRP109, GLN113 TYR152	MET58, PRO81, TYR83, ILE111, TYR152	TYR152	
**4**-3HY3	-	-	PRO81, TYR83, ILE111	-	TYR83
**5**-3HY3	-	THR107, TYR152	TYR83, PHE85 TRP109	-	TYR23 TYR128
**6**-3HY3			MET58, PRO81, TYR83, ILE111	LEU56, PRO81 PRO112, GLN113	
**7**-3HY3			PHE55	GLU61, THR107 ILE111, PRO112 GLN113, TYR152	
5-FU-3HY3		TRP109, TYR152	PRO8		
**2**-6ECZ	THR199	HIS119	HIS94, HIS119, VAL121 VAL143, LEU198, PRO202	-	
**3**-6ECZ		HIS94	HIS94, HIS119, PHE131 LEU198, PRO202, RP209	THR200	
**4**-6ECZ	THR200	-	HIS94, HIS119 LEU198	-	HIS94 TRP209
**5**-6ECZ	-	HIS94, THR200	HIS94, HIS96, VAL143, LEU198, TRP209	-	TRP209
**6**-6ECZ			LEU198, HIS94 VAL121	THR199, SER197	
**7**-6ECZ			PHE231, HIS64	ASN62, GLY63	
5-FU-6ECZ		HIS94	PHE131, PRO202, TRP209		
**2**-6FL5	-	-	PHE125, LEU149, LE160, ALA162	THR163	
**3**-6FL5	-	SER53, ASN387 THR388, ARG402	LEU143, CYS204	LEU143, GLY147	
**4**-6FL5	-	ASN387, THR388	LEU143, CYS204	-	
**5**-6FL5	-	HIS94, THR200	HIS94, HIS96, VAL143, LEU198, TRP209	-	PHE125
**6**-6FL5			ALA162, PHE125	SER121, THR163	
**7**-6FL5			ALA162, LEU149	THR163	
5-FU-6FL5		ASN387	ILE160		
