# Supplementary figures and images for: Bromination and conversion of tetrahydro-1H-indene to bisoxirane with a new approach: synthesis, structural characterization by spectroscopic and theoretical methods, and biological analysis supported by DFT and docking
Source: Turk J Chem. 2023 Oct 11;47(6):1459–78. doi: 10.55730/1300-0527.3628 (PMC10965191; doi:10.55730/1300-0527.3628)

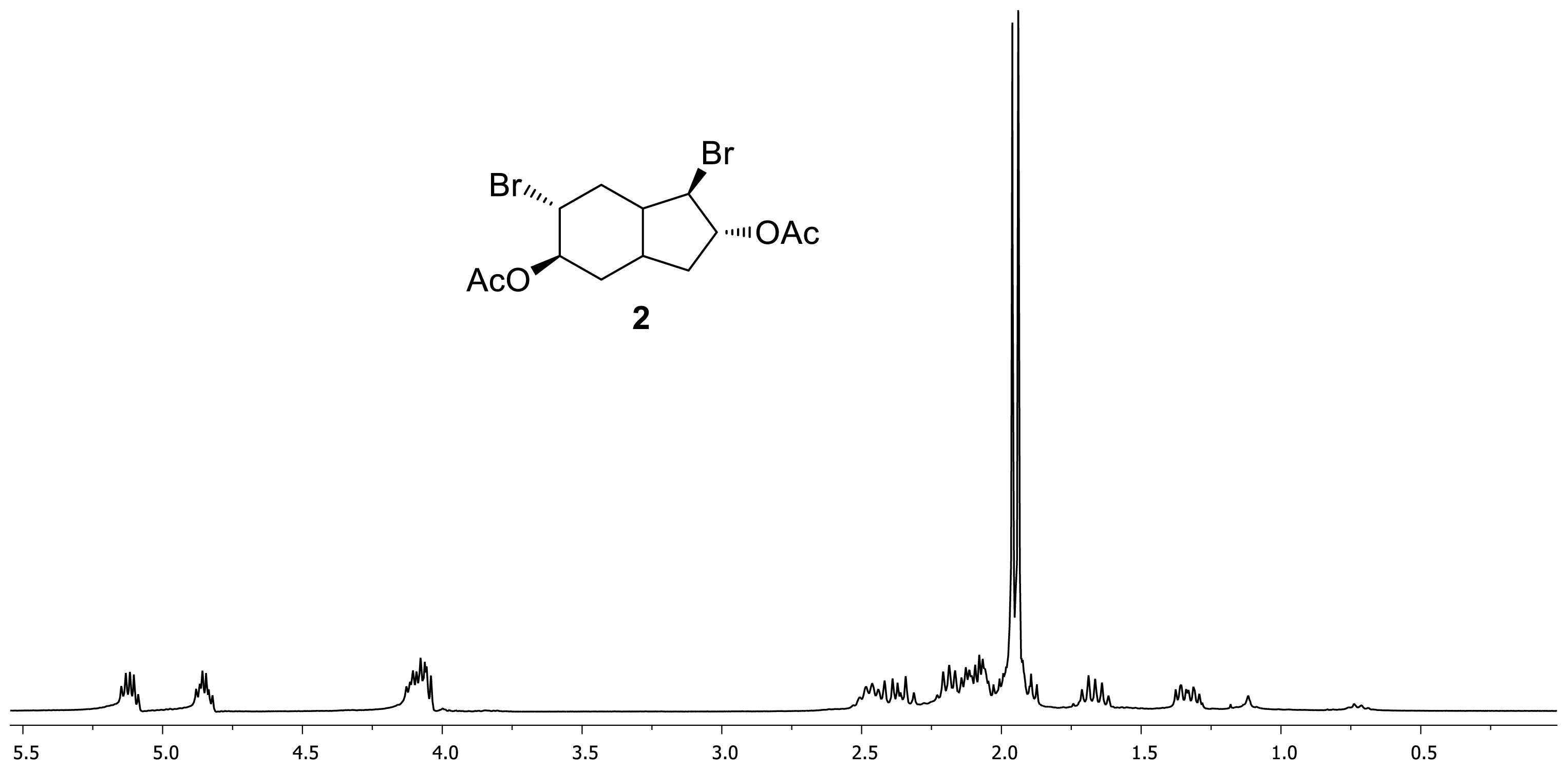

Supplement: Figure 1S — 1H NMR spectrum of dibromodiacetate 2 (300 MHz, in CDCl3). [file tjc-47-06-1459s1.tif]

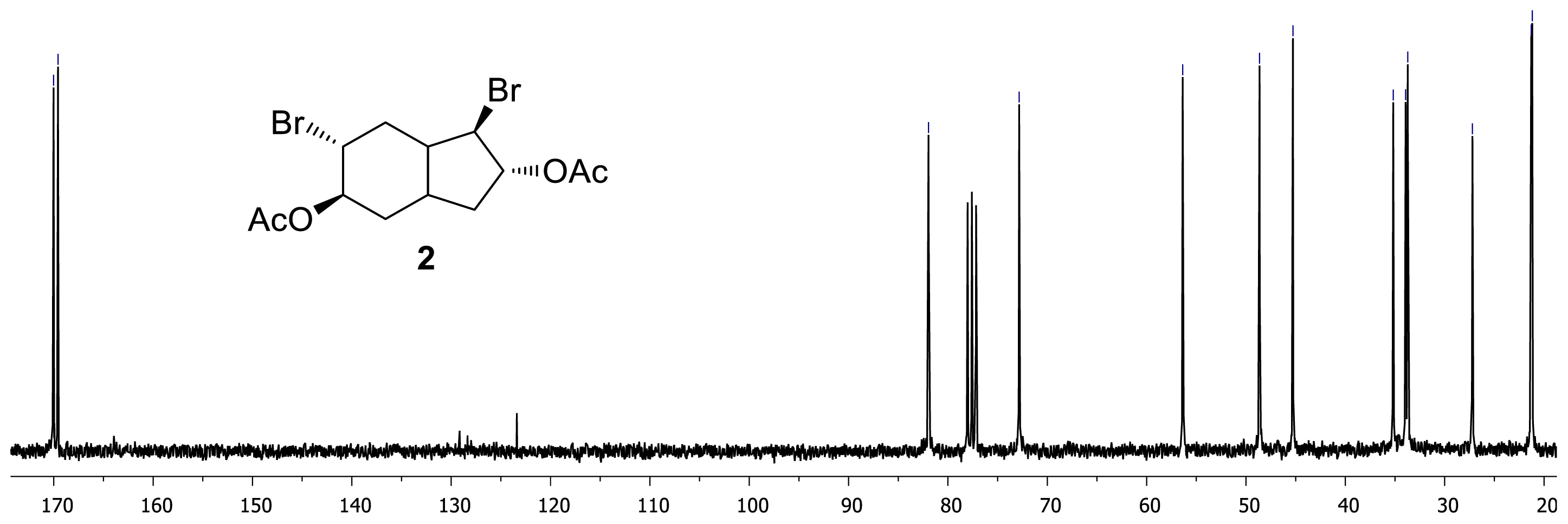

Supplement: Figure 2S — 13C NMR spectrum of dibromodiacetate 2 (75 MHz, in CDCl3). [file tjc-47-06-1459s2.tif]

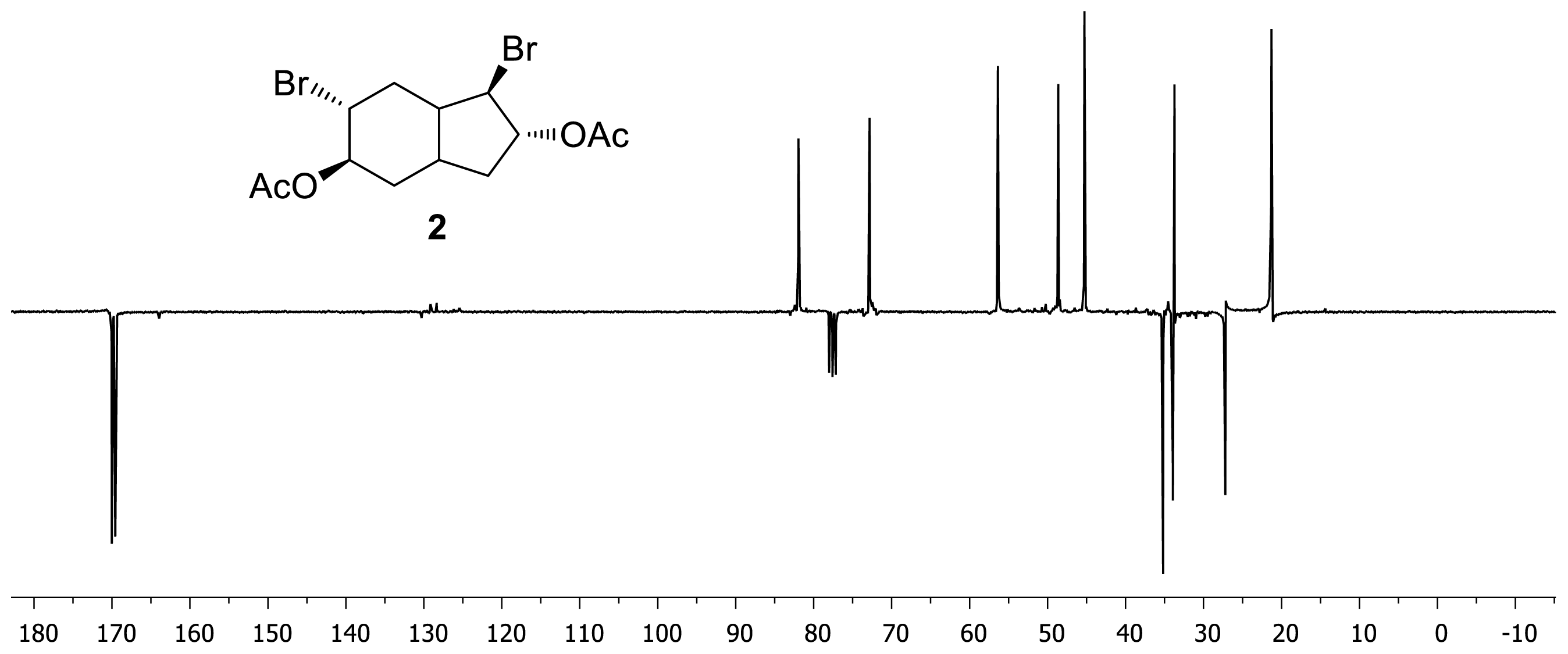

Supplement: Figure 3S — APT spectrum of dibromodiacetate 2. [file tjc-47-06-1459s3.tif]

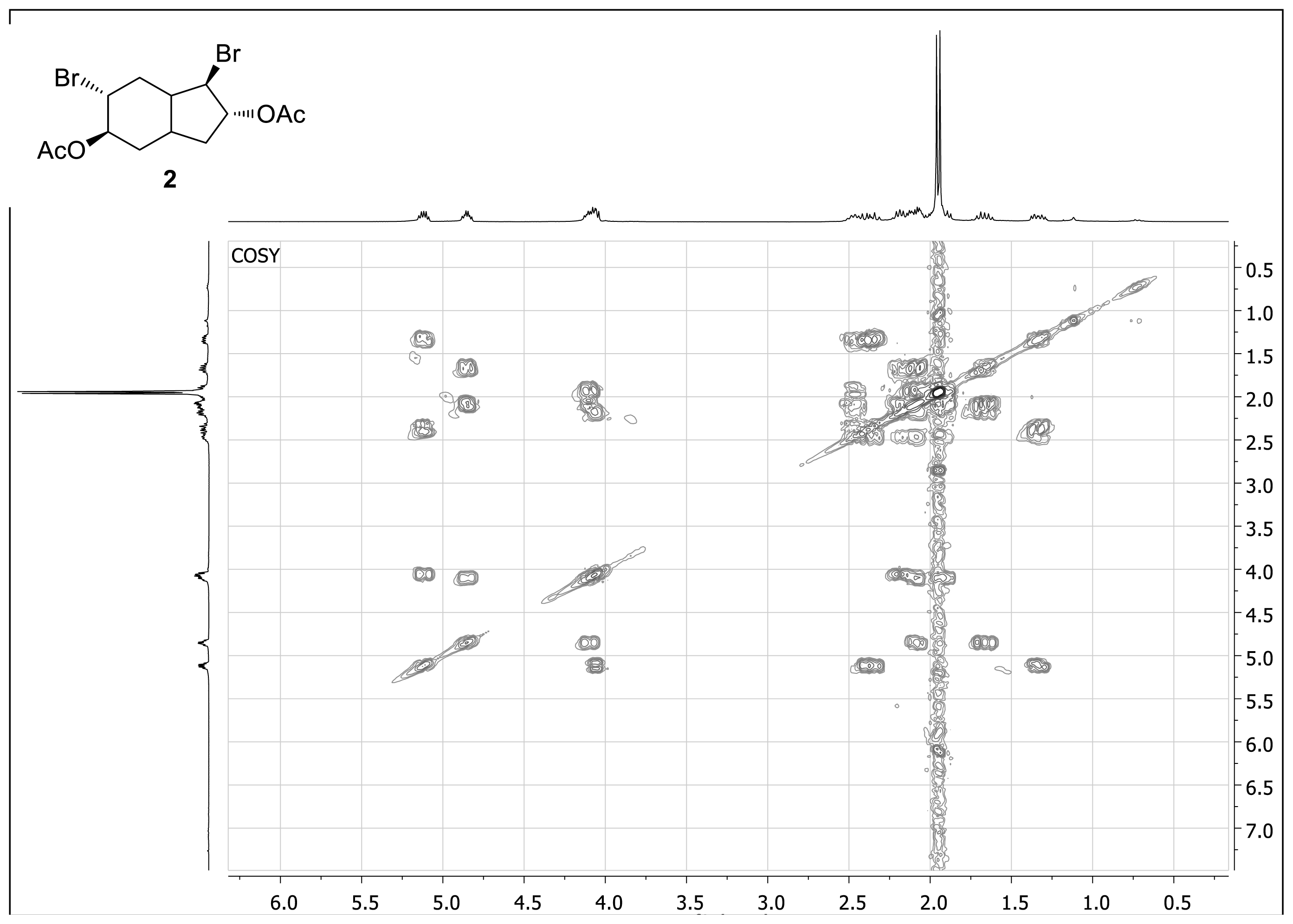

Supplement: Figure 4S — COSY spectrum of dibromodiacetate 2. [file tjc-47-06-1459s4.tif]

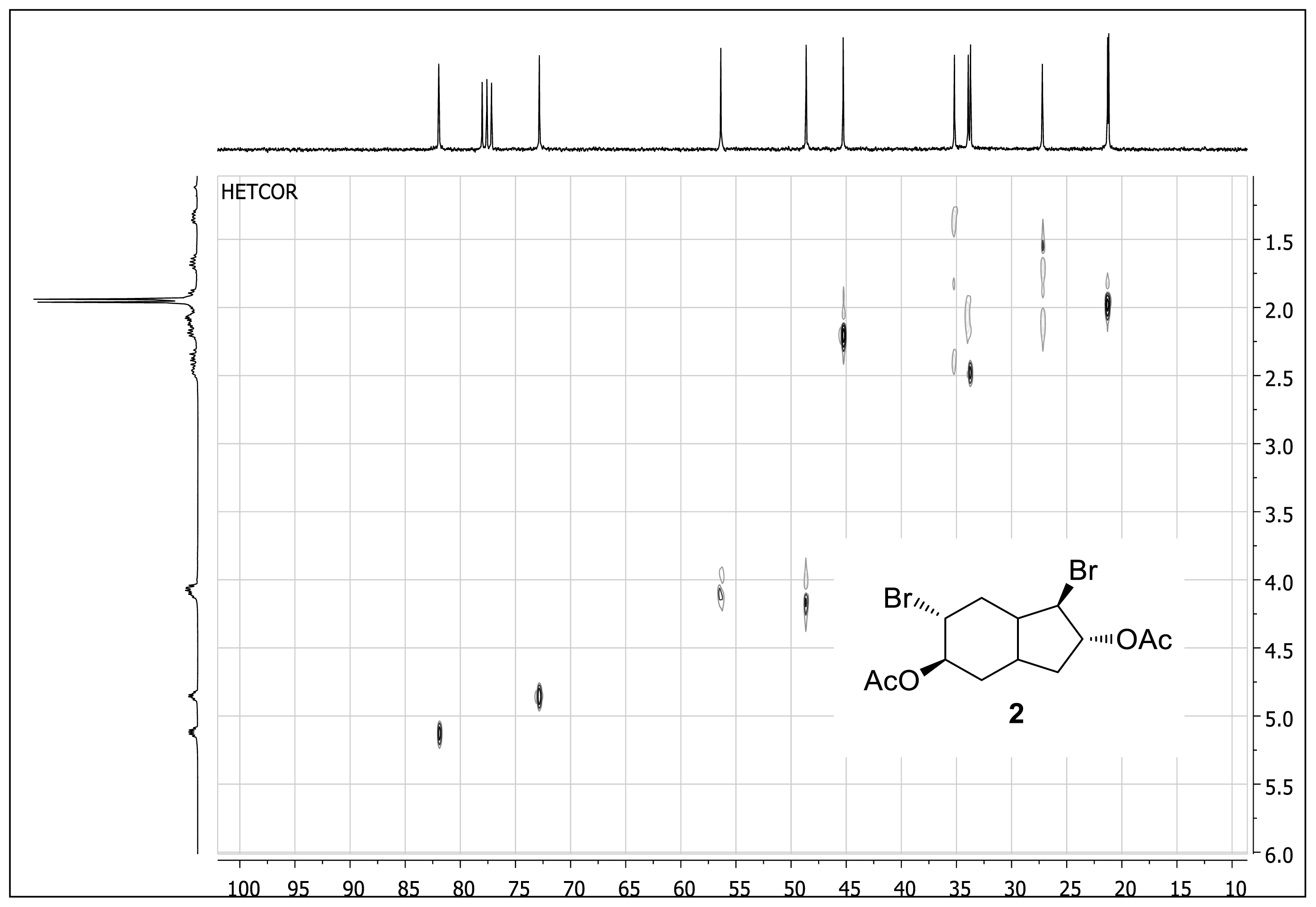

Supplement: Figure 5S — HETCOR spectrum of dibromodiacetate 2. [file tjc-47-06-1459s5.tif]

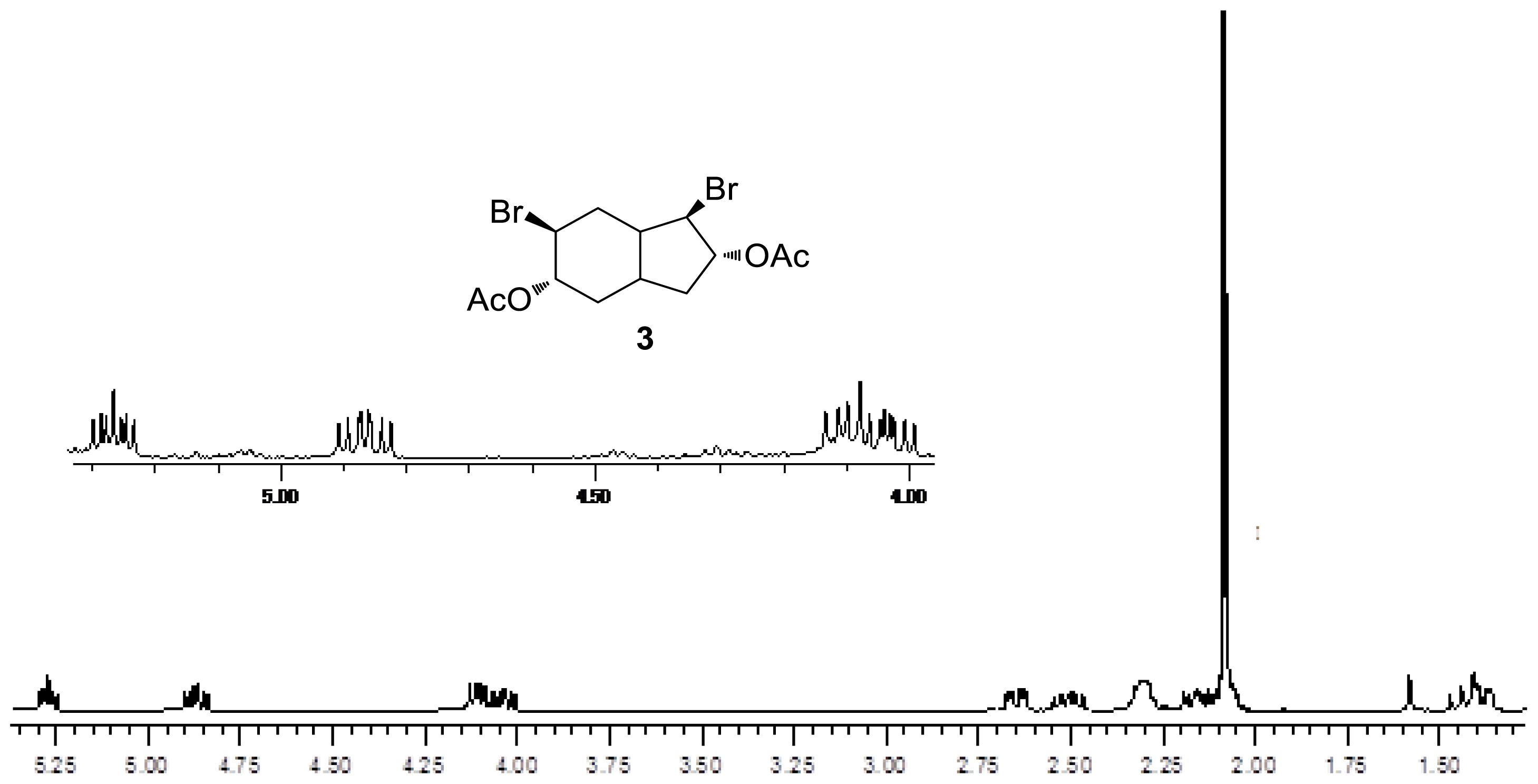

Supplement: Figure 6S — 1H NMR spectrum of dibromodiacetate 3 (300 MHz, in CDCl3). [file tjc-47-06-1459s6.tif]

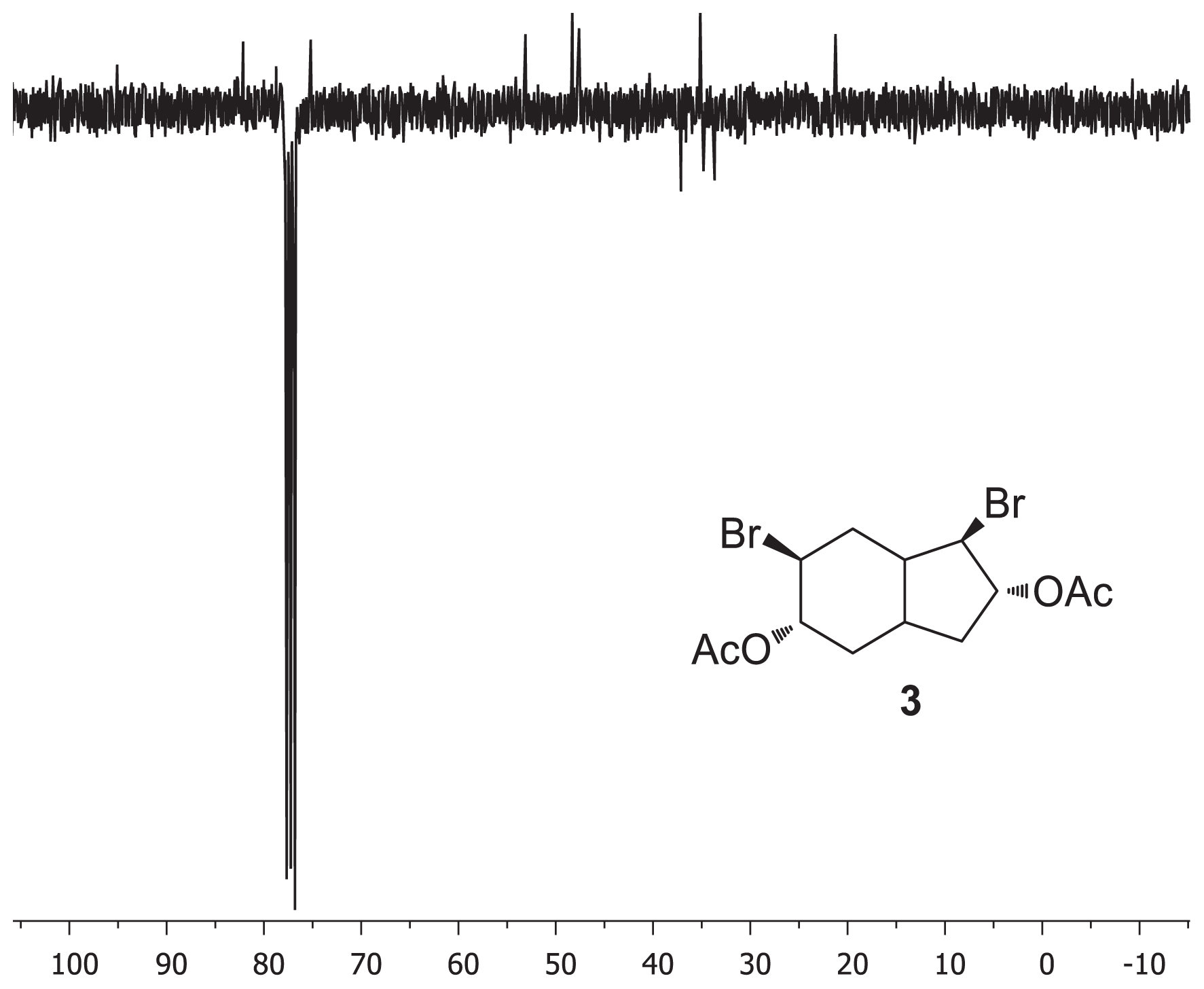

Supplement: Figure 7S — APT spectrum of product dibromodiacetate 3. [file tjc-47-06-1459s7.tif]

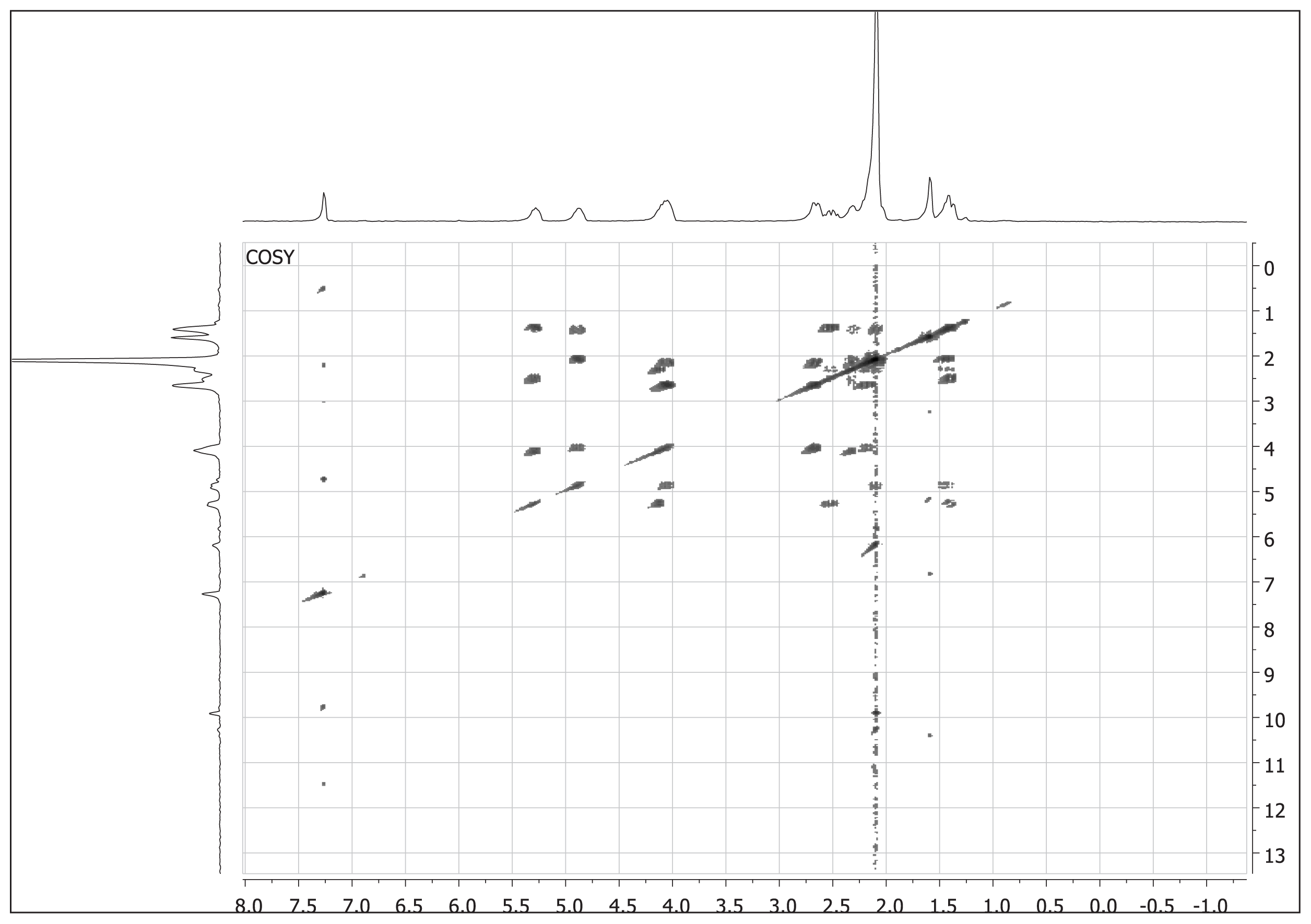

Supplement: Figure 8S — COSY spectrum of product dibromodiacetate 3. [file tjc-47-06-1459s8.tif]

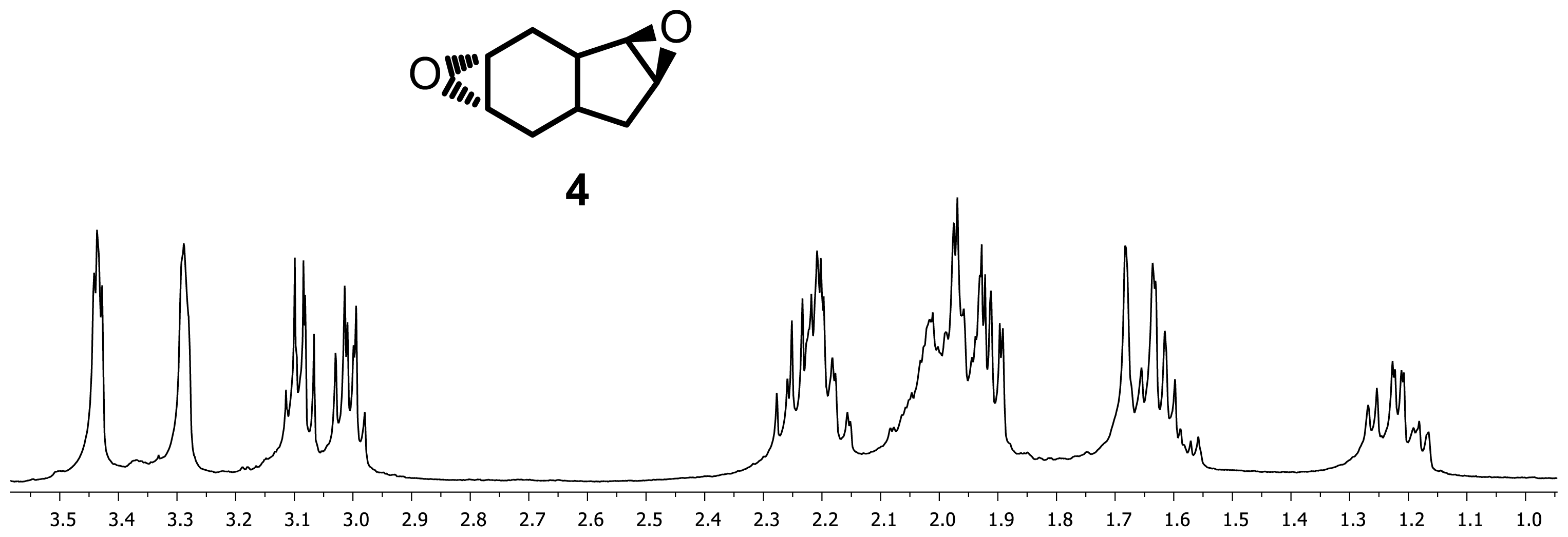

Supplement: Figure 9S — 1H NMR spectrum of diepoxide 4. [file tjc-47-06-1459s9.tif]

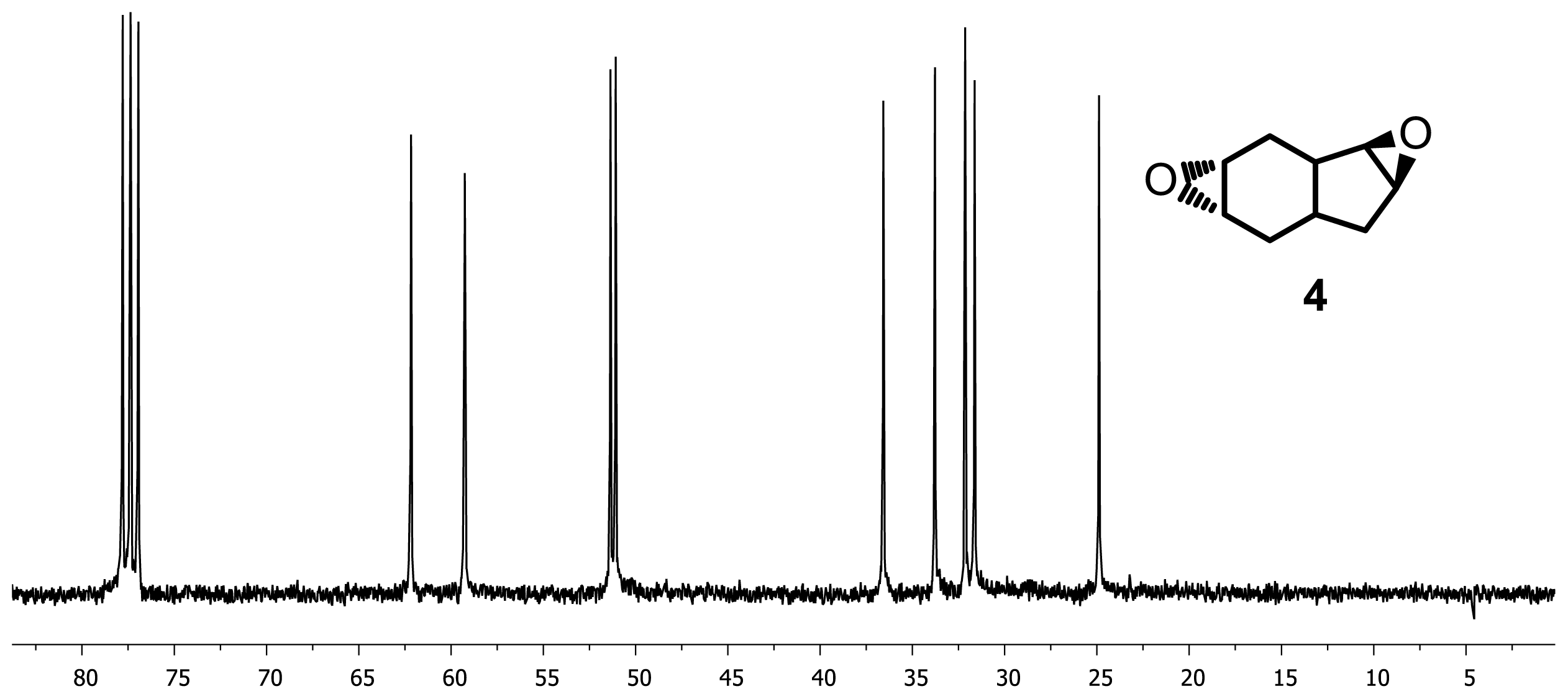

Supplement: Figure 10S — 13C NMR spectrum of diepoxide 4 (75 MHz, in CDCl3). [file tjc-47-06-1459s10.tif]

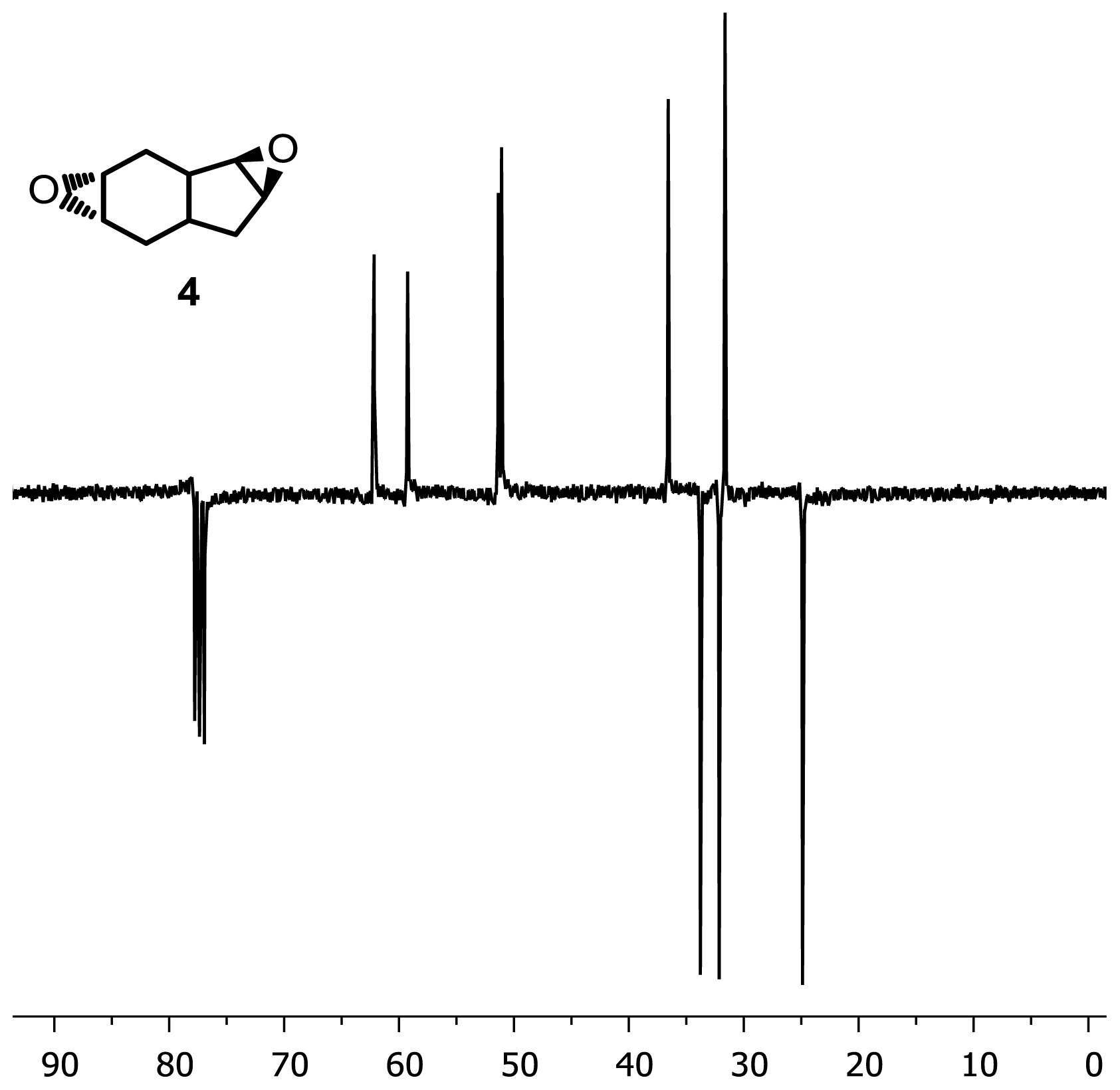

Supplement: Figure 11S — APT spectrum of diepoxide 4. [file tjc-47-06-1459s11.tif]

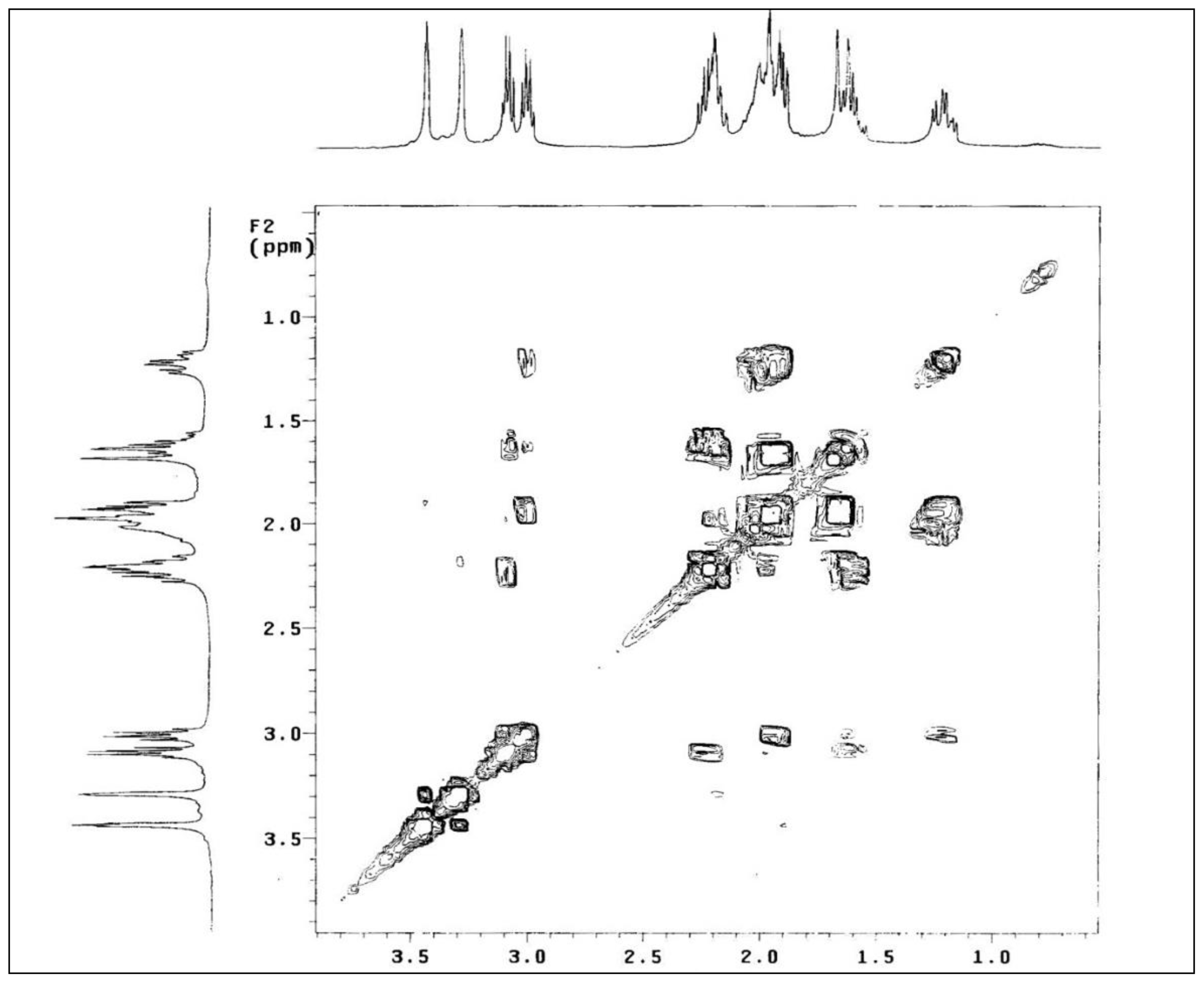

Supplement: Figure 12S — COSY spectrum of diepoxide 4. [file tjc-47-06-1459s12.tif]

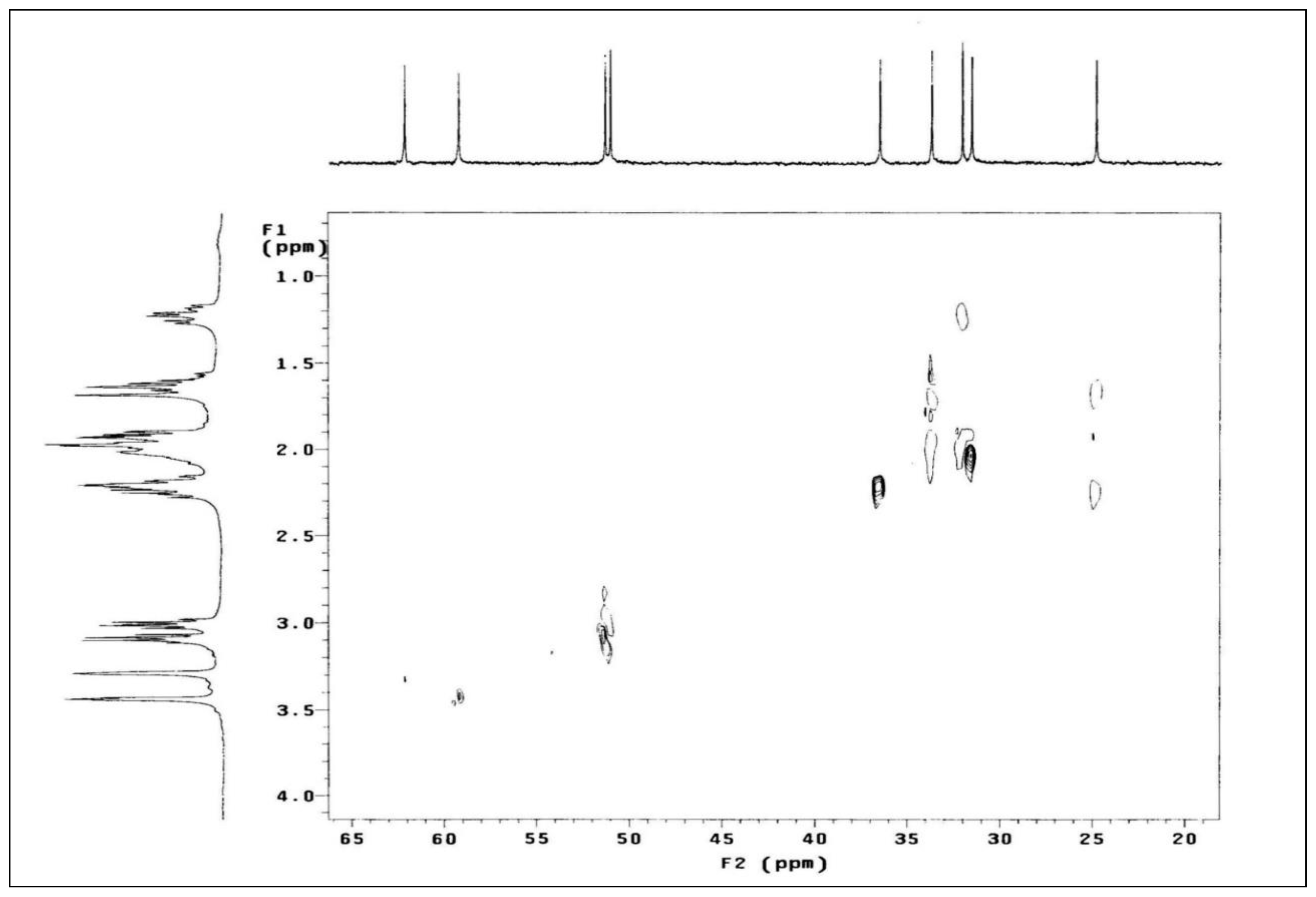

Supplement: Figure 13S — HETCOR spectrum of product diepoxide 4. [file tjc-47-06-1459s13.tif]

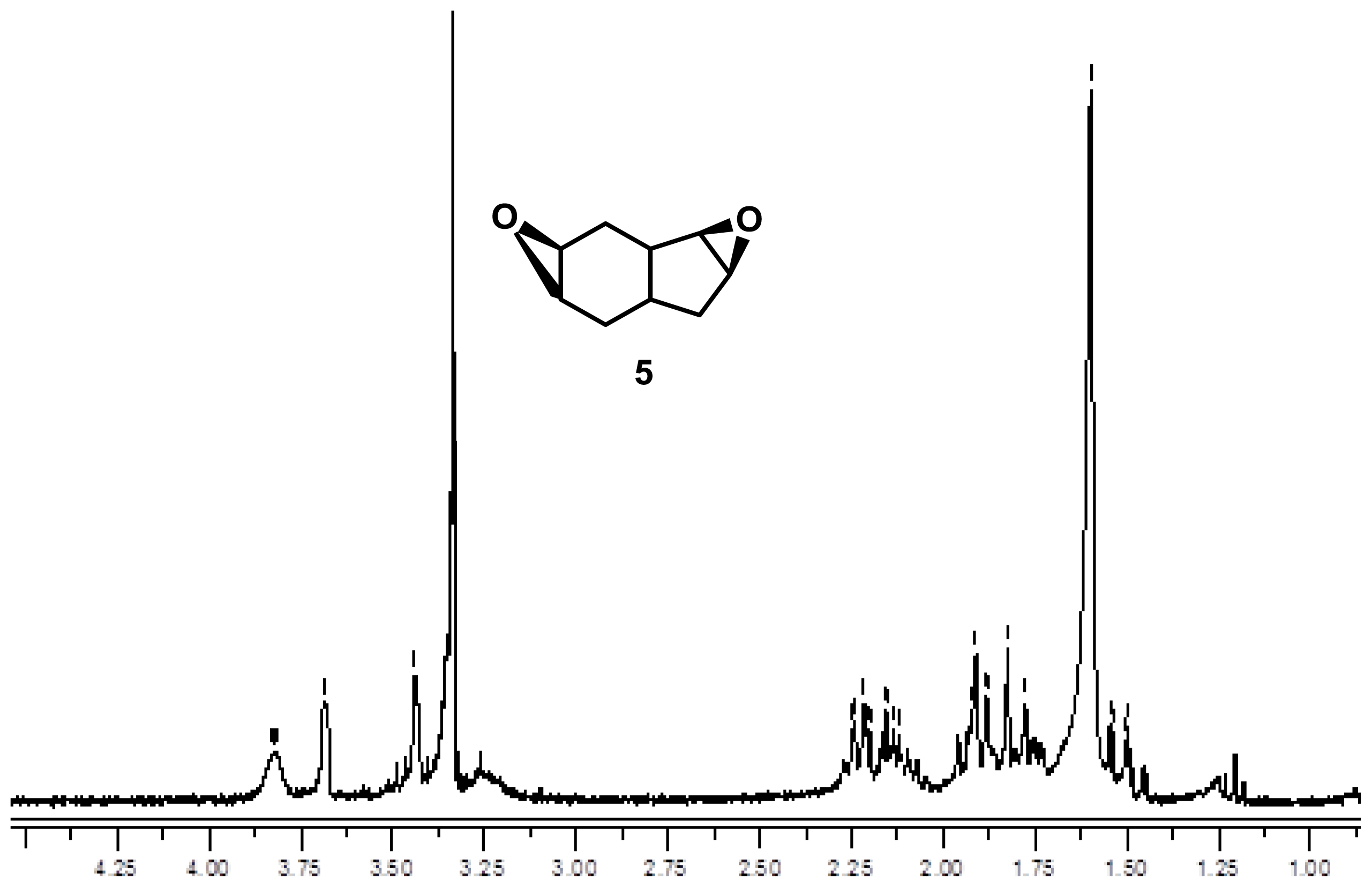

Supplement: Figure 14S — 1H NMR spectrum of product diepoxide 5 (300 MHz, in CDCl3). [file tjc-47-06-1459s14.tif]

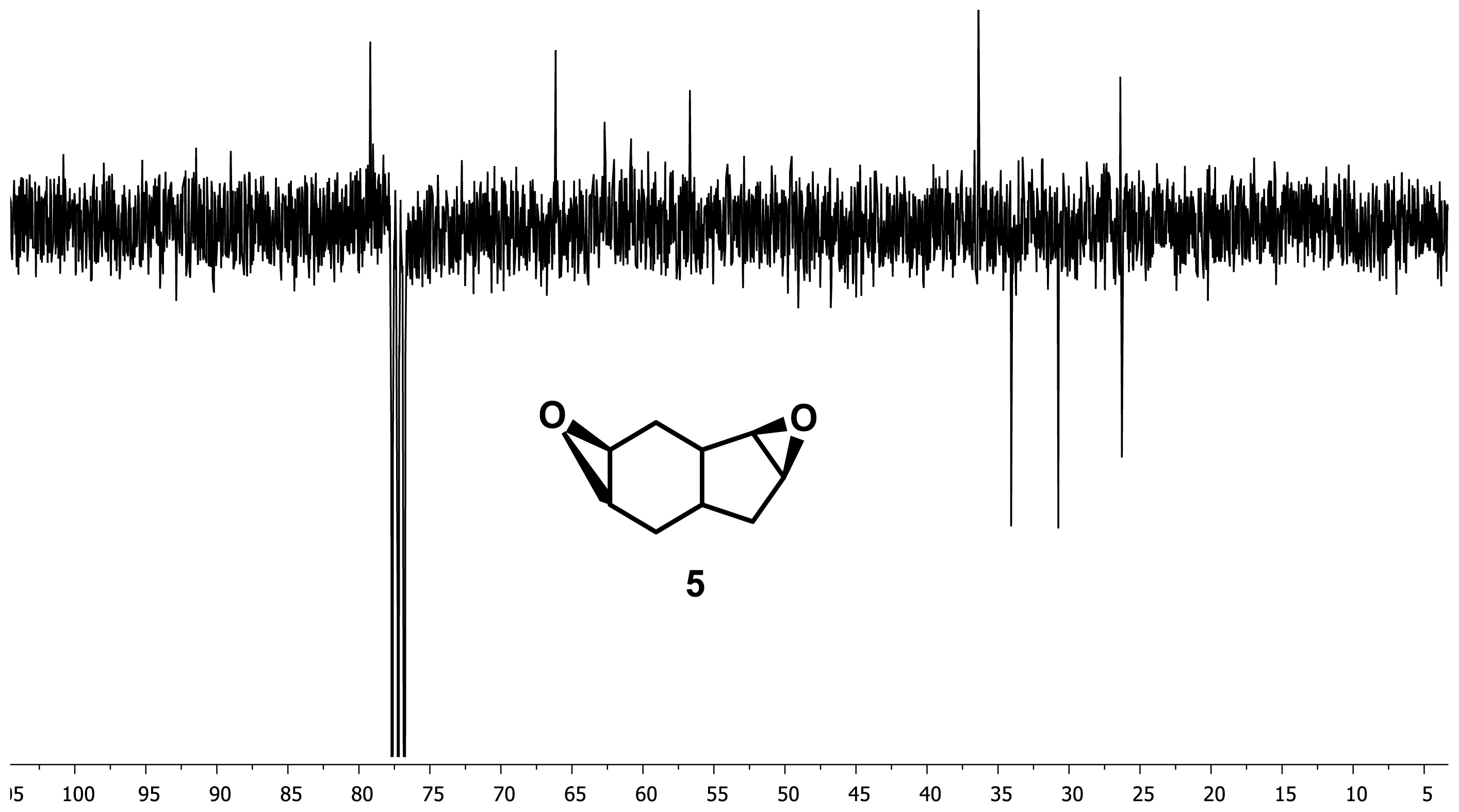

Supplement: Figure 15S — APT spectrum of diepoxide 5. [file tjc-47-06-1459s15.tif]

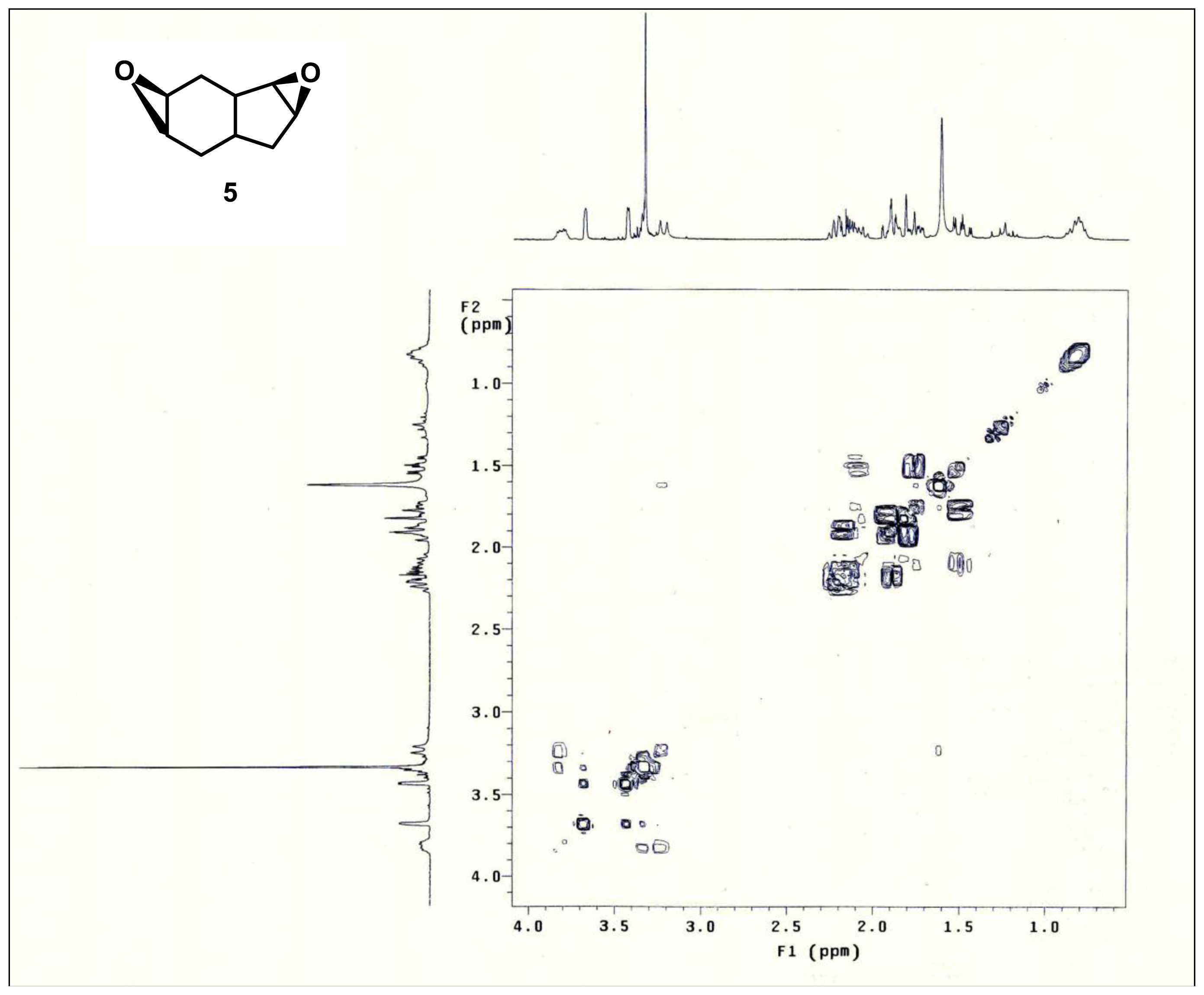

Supplement: Figure 16S — COSY spectrum of diepoxide 5. [file tjc-47-06-1459s16.tif]

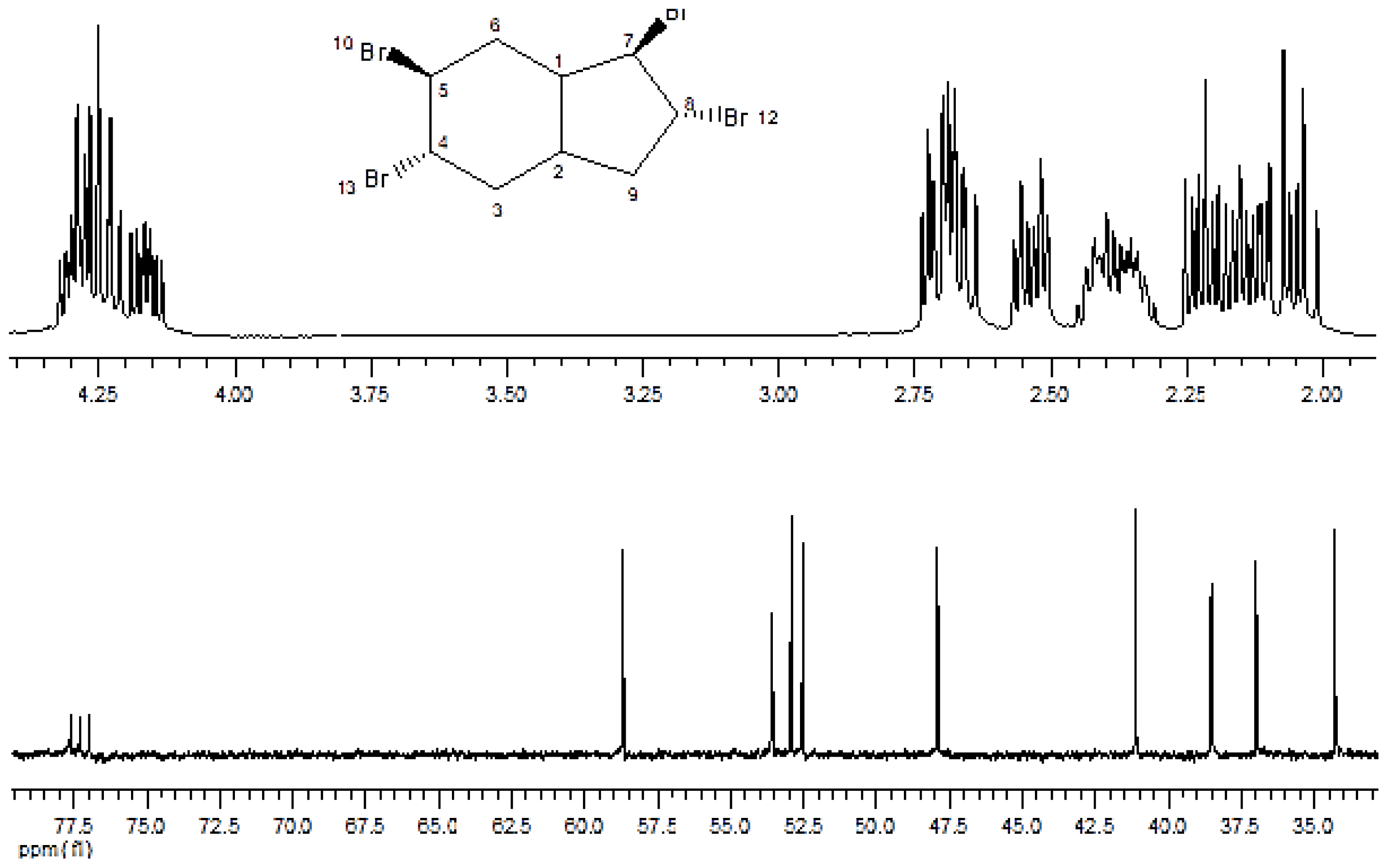

Supplement: Figure 17S — 1H NMR and 13C NMR spectra of tetrabromide 6 (300/75 MHz, in CDCl3). [file tjc-47-06-1459s17.tif]

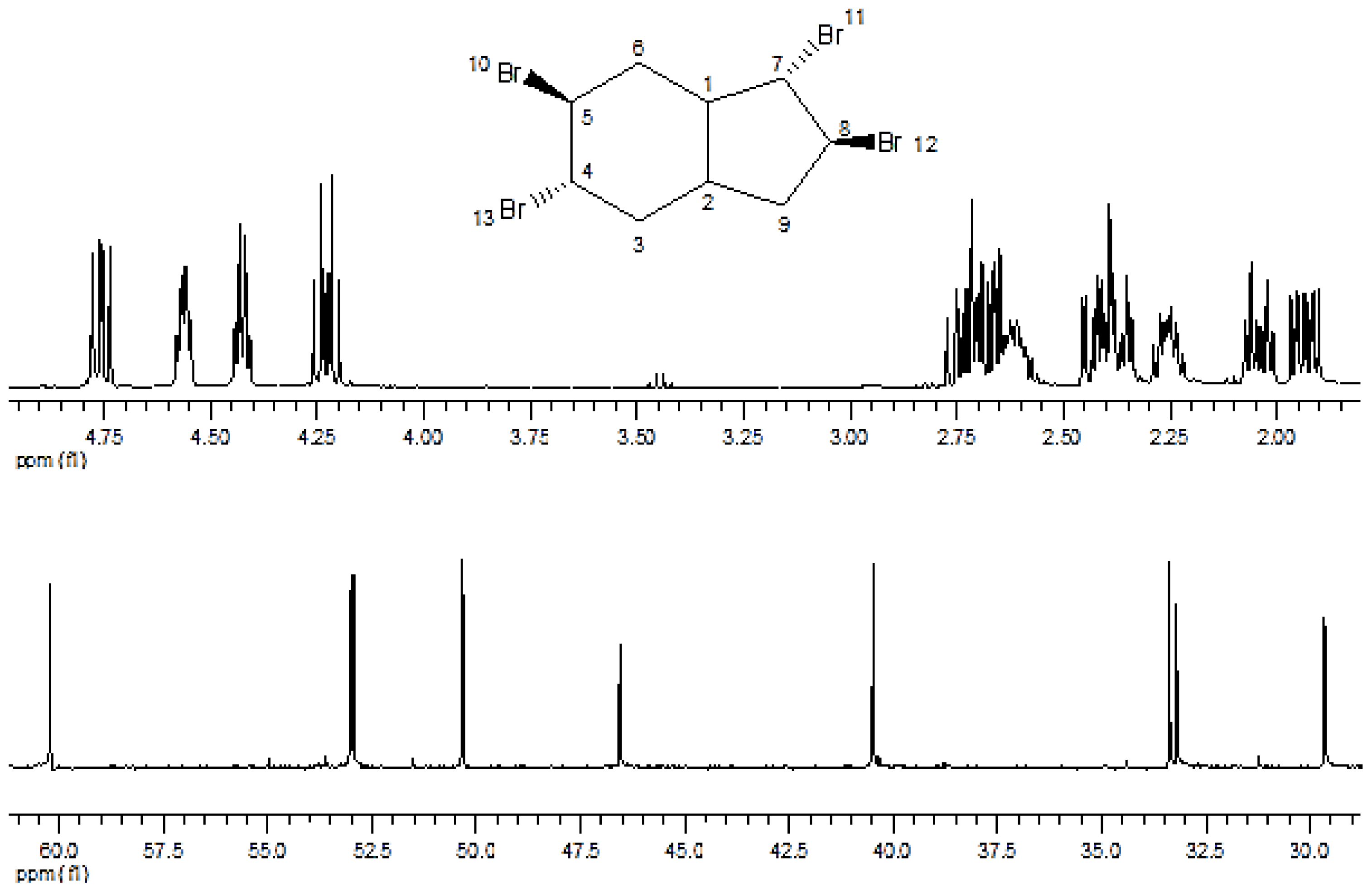

Supplement: Figure 18S — 1H NMR and 13C NMR spectra of tetrabromide 7 (300/75 MHz, in CDCl3). [file tjc-47-06-1459s18.tif]
